# Marizomib suppresses triple-negative breast cancer via proteasome and oxidative phosphorylation inhibition

**DOI:** 10.7150/thno.42705

**Published:** 2020-04-06

**Authors:** Prahlad V. Raninga, Andy Lee, Debottam Sinha, Lan-feng Dong, Keshava K. Datta, Xue Lu, Priyakshi Kalita-de Croft, Mriga Dutt, Michelle Hill, Normand Pouliot, Harsha Gowda, Murugan Kalimutho, Jiri Neuzil, Kum Kum Khanna

**Affiliations:** 1 QIMR Berghofer Medical Research Institute, 300 Herston Road, Herston, Brisbane QLD 4006, Australia; 2 Radiation Biology Research Center, Institute for Radiological Research, Chang Gung Memorial Hospital/Chang Gung University, Taoyuan 333, Taiwan; 3 School of Medical Science, Griffith University, Southport, QLD 4222, Australia; 4 UQ Centre for Clinical Research, Faculty of Medicine, The University of Queensland, Herston, QLD 4029, Australia; 5 Olivia Newton-John Cancer Research Institute, 145 Studley Road, Heidelberg Vic 3084 Australia; 6 Institute of Biotechnology, Czech Academy of Sciences, Prague-West, 252 50 Czech Republic

**Keywords:** Marizomib, triple-negative breast cancer, metastasis, oxidative phosphorylation, glycolysis

## Abstract

**Purpose**: Lacking effective targeted therapies, triple-negative breast cancer (TNBCs) is highly aggressive and metastatic disease, and remains clinically challenging breast cancer subtype to treat. Despite the survival dependency on the proteasome pathway genes, FDA-approved proteasome inhibitors induced minimal clinical response in breast cancer patients due to weak proteasome inhibition. Hence, developing effective targeted therapy using potent proteasome inhibitor is required.

**Methods**: We evaluated anti-cancer activity of a potent proteasome inhibitor, marizomib, *in vitro* using breast cancer lines and *in vivo* using 4T1.2 murine syngeneic model, MDA-MB-231 xenografts, and patient-derived tumor xenografts. Global proteome profiling, western blots, and RT-qPCR were used to investigate the mechanism of action for marizomib. Effect of marizomib on lung and brain metastasis was evaluated using syngeneic 4T1BR4 murine TNBC model *in vivo*.

**Results**: We show that marizomib inhibits multiple proteasome catalytic activities and induces a better anti-tumor response in TNBC cell lines and patient-derived xenografts alone and in combination with the standard-of-care chemotherapy. Mechanistically, we show that marizomib is a dual inhibitor of proteasome and oxidative phosphorylation (OXPHOS) in TNBCs. Marizomib reduces lung and brain metastases by reducing the number of circulating tumor cells and the expression of genes involved in the epithelial-to-mesenchymal transition. We demonstrate that marizomib-induced OXPHOS inhibition upregulates glycolysis to meet the energetic demands of TNBC cells and combined inhibition of glycolysis with marizomib leads to a synergistic anti-cancer activity.

**Conclusions**: Our data provide a strong rationale for a clinical evaluation of marizomib in primary and metastatic TNBC patients.

## Introduction

Triple-negative breast cancers (TNBCs), defined by their lack of estrogen (ER), progesterone (PR), and Her2 receptors, are a very aggressive type of breast cancer (BC) that typically affects young women and has the worst prognosis of any BC subtype. Although a majority of TNBC patients respond to initial chemotherapies, these tumors are prone to recur, metastasize (disseminate to the lungs and brain), and acquire chemoresistance 1. TNBCs are enriched for poorly differentiated tumor initiating cells, which are often responsible not only for tumor initiation but also for metastasis and chemoresistance. Thus, there is an urgent need to explore clinically viable options for treatment of primary and metastatic TNBCs.

Proteasome inhibitors have been shown to exert a significant anti-cancer activity in multiple cancer types including multiple myeloma and leukemia. A genome-wide siRNA screen identified the survival dependency of TNBC compared to other breast cancer subtypes on proteasome genes 2, suggesting that the proteasome complex represents a plausible therapeutic target in TNBCs due to disrupted proteostasis contributed by their inherent genomic instability 3. Although a first-generation FDA-approved proteasome inhibitor, bortezomib (Btz), and second-generation inhibitors including carfilzomib (Cfz) and ixazomib (Ixz) induced a significant anti-tumor activity in pre-clinical models 2, 4, they have not demonstrated efficacy in clinical trials in solid tumors including TNBCs, either alone or in combination with other therapies 4. This could be due to weaker proteasome inhibitory activity of these drugs in solid tumors. Furthermore, as a single agent, carfilzomib showed no tumor suppressive effect in an MDA-MB-231 xenograft model 5.

The 26S proteasome is composed of a cylindrical 20S core that is capped by the regulatory 19S component 6. The 20S core contains three proteasome active-site subunits within its β rings including the β1 site (caspase-like), β2 site (trypsin-like), and β5 site (chymotrypsin-like) 7. While Btz, Cfz, and Ixz selectively inhibit the chymotrypsin-like proteasome activity (CT-L) 8, they fail to inhibit the trypsin-like (T-L) and caspase-like (CP-L) proteasome activity, which are known to degrade proteins 9. A recent study has shown that Btz/Cfz-induced CT-L proteasome activity inhibition resulted in upregulation of T-L and CP-L proteasome activity in Nrf1-dependent manner 10, which is a dominant resistance mechanism to these drugs. Notably, selective inhibition of T-L (β2) proteasome activity sensitized TNBC cells to Btz/Cfz-induced apoptosis 10. Hence, co-inhibition of multiple active sites of the proteasome complex could increase the potency and anti-tumor activity of proteasome inhibitors in solid tumors including TNBCs.

Marizomib (Mzb), also known as NPI-0052, is an orally active, small molecule proteasome inhibitor derived from marine bacteria 11. Mzb has been shown to irreversibly inhibit all three proteasome active sites (CT-L, T-L, and CP-L) in multiple myeloma and in solid tumors 8, 12, thus has increased potency to be a multisite antagonist of the proteasome complex compared to other peptide-based proteasome inhibitors. Unlike other proteasome inhibitors, Mzb is capable of crossing the blood-brain barrier and exerted a significant anti-tumor activity in glioblastoma 13, suggesting that Mzb may exert a significant effect on brain metastatic TNBCs. Mzb is currently being tested in phase I, II, and III clinical trials for refractory multiple myeloma, leukemia, lymphoma, glioblastoma, and malignant glioma, both as a single agent and in combination therapies. The results from clinical trials show that Mzb, both as a monotherapy and in combination with pomalidomide, is well-tolerated and demonstrates promising activity in relapsed and refractory multiple myeloma 14. Based on this information, we hypothesized that Mzb can simultaneously inhibit multiple proteasome catalytic sites, which may enhance its anti-tumor activity in TNBCs compared to the previous generation compounds.

In this study we examined the anti-cancer activity of Mzb as a monotherapy in both primary and metastatic TNBCs. We show that Mzb strongly inhibits proteasome functions by blocking CT-L, T-L, and CP-L proteasome activity, induces caspase-3 dependent apoptosis, and inhibits tumor cell proliferation *in vitro* in 2D and 3D cultures. Here, we describe an additional, novel mechanism for the anti-cancer activity of Mzb and demonstrate that Mzb inhibits complex II-dependent mitochondrial respiration leading to a reduced oxidative phosphorylation (OXPHOS). We further show that Mzb significantly reduces primary tumor growth and induces apoptosis in human TNBC cell line xenografts, a murine syngeneic TNBC model, and patient-derived tumor xenograft (PDX) models *in vivo*. Mzb also exerts a synergistic anti-cancer activity in combination with doxorubicin *in vivo.* Our results document that Mzb inhibits TNBC metastasis by inhibiting OXPHOS and reducing the number of circulating tumor cells (CTCs) *in vivo.* Finally, we show that Mzb significantly reduces lung and brain metastatic burden *in vivo* after resecting the primary TNBC tumors.

## Materials and Methods

### Cell lines and reagents

All breast cancer cell lines and non-malignant mammary epithelial cell line (MCF10A) were obtained from the American Type Culture Collection (ATCC). The 4T1.2 and 4T1BR4 cell lines were kindly provided by Dr Norman Pouliot, Olivia Newton-John Cancer Research Institute, Australia. All breast cancer cell lines were cultured in DMEM media supplemented with 10% fetal bovine serum (FBS). D492 mammary epithelial cells were provided by Dr Thorarinn Gudjonsson (The Panum Institute, Denmark) and were maintained as described previously 15. All cell lines were tested for Mycoplasma infection and authenticated using short tandem repeat (STR) profiling by scientific services at QIMR Berghofer Medical Research Institute. Marizomib was purchased from Cayman Chemicals (Cat #: 10007311). STF-31 was purchased from Selleck Chemicals (Cat #: S7931). The list of antibodies and RT-qPCR primers used in this study is provided in Table S1 and S2, respectively.

### ROUTINE Respiration

SUM159PT or MDA-MB-231 were grown to 50-70% confluence, treated with either Mzb or Btz (100 nM) for 9 h and 24 h, respectively. Cells were harvested and suspended in DMEM medium without serum. Oxygen consumption for ROUTINE respiration, oligomycin-inhibited LEAK respiration, FCCP-stimulated uncoupled respiration capacity (ETS) and rotenone/antimycin-inhibited residual respiration (ROX) were evaluated using the Oxygraph-2k instrument (Oroboros) on intact cells. The results were normalized to the cell numbers.

### Mitochondrial Respiration

SUM159PT or MDA-MB-231 were grown to 50-70% confluence, treated with Mzb (100 nM) for 9 h and 24 h, respectively. Cells were harvested and suspended in mitochondrial respiration medium MiR06, and then permeabilized by digitonin. Oxygen consumption was evaluated using the Oxygraph-2k instrument for Complex I/II-linked respiration in the presence of the proper substrates and inhibitors of the other complex. The results were normalized to the cell numbers.

### *In vivo* xenografts and tumor growth analysis

All experiments were conducted in accordance to the guidelines of the QIMR Berghofer Medical Research Institute Animal Ethics Committee and as described previously 16. For human cell line MDA-MB-231 xenograft model, 3 X 10^6^ cells were prepared in 50% growth factor-reduced Matrigel (BD, Biosciences, Bedford, USA)/PBS and injected into the right 4^th^ inguinal mammary fat pad of 6-weeks old female immunocompromised NOD/SCID mice. For murine 4T1.2 and 4T1BR4 syngeneic models, 1 X 10^5^ cells prepared in 1X PBS were injected into the right 4^th^ inguinal mammary fat pad of 6-weeks old female immunocompetent Balb/c mice. For the patient-derived tumor xenograft (PDX) model, 2 mm tumor biopsy of the TNBC patient (coded TNBC0019) was implanted into the right 4^th^ inguinal mammary fat pad of 6-weeks old female immunocompromised NOD/SCID mice. Passaging of PDX tumors of breast cancer was conducted according to the IRB guidelines of Chang Gung Memorial Hospital, Taoyuan, Taiwan. Once tumor size reached ~30-50 mm^3^, mice were randomized blindly into different treatment groups and were then treated with the vehicle or Mzb (0.15 mg/kg, twice per week, IP) for 2 weeks. Tumor growth was measured thrice weekly using a digital caliper. To calculate the tumor volume the following formula was used: tumor volume = [Lx W^2^]/2, where W = width of the tumor and L = length of the tumor.

For marizomib and 2-deoxy-D-glycose (2-DG) combination therapy, mice were treated with either vehicle, Mzb (half MTD of 0.075 mg/kg, twice/week), 2-DG (400 mg/kg, IP, 3 times/week), and combination for 2 weeks. For Mzb and doxorubicin combination therapy, mice were treated with the vehicle, Mzb (0.075 mg/kg, twice/week), doxorubicin (10 mg/kg, IP, once a week), and combination for 2 weeks.

### Flow cytometry analysis of ALDH1^+^ stem-cell population

PDX tumors were mashed, filtered through a 100μm mesh, and washed with PBS. After washing in RPMI medium, live cells in the interphase were collected from Ficoll solution and treated with DNase I (0.02 mg/ml), hyaluoronidase type V (0.2 mg/ml), collagenase 4 (0.5 mg/ml) at 37ºC for 2 h. Cells were washed in PBS with 5% FBS and subjected to RBC lysis before single cell suspensions were obtained from TryPLE solution (Gibco). ALDH1 positive populations were determined by conducting ALDEFLUOR^TM^ assay following manufactures' instruction (StemCell Technologies, MA, USA). Data acquisition and analysis were performed using NovoCyte Flow Cytometer (ACEA Biosciences, CA, USA).

### *In vivo* metastasis analysis - resection model

*In vivo* metastasis experiments were conducted in accordance to the guidelines of the QIMR Berghofer Animal Ethics Committee. For murine 4T1BR4 syngeneic model, 1 X 10^5^ cells were suspended in 1X PBS and injected into the 4^th^ inguinal mammary fat pad of 6-weeks old female Balb/c mice. Once tumor size reached ~250-300 mm^3^, the primary tumors were resected. Two days after the tumor resection, mice were treated with the vehicle or Mzb (0.15 mg/kg, twice per week, IP) for 2 weeks. At the end of two weeks treatment, lungs and brain were excised. For lung metastasis, lungs were perfused with 1X PBS to remove residual blood. Both lungs and brain were fixed with 10% formalin and stained with hematoxylin and eosin (H&E) to facilitate counting of micro- and macro-metastatic nodules.

### Isolation of circulating tumor cells

Post 1-week drug treatment, mice were euthanized and 100µl of blood was collected from the left ventricle into Eppendorf tubes containing 100µl of heparin solution to avoid blood clotting, and kept on ice. The samples were centrifuged at 400 x g for 5 min at 4⁰C. The serum was discarded and the cell pellet was re-suspended in 1 ml of red blood cell lysis buffer (150 mM NH_4_Cl, 10 mM KHCO_3_, and 0.1 mM EDTA) before centrifugation at 400 x g for 5 min at 4⁰C. The supernatant was discarded and the cell pellet was re-suspended with 1 ml of cold PBS before subjection to several centrifugation cycles to remove debris and hemoglobin from the tumor cells and lymphocytes. The resulting pellet was resuspended in 500 µl of complete growth media and 50 µl of the tumor cell suspension was transferred into 60 mm culture dishes, and incubated for 14 days. Circulating tumor cell colonies that formed after 14 days were fixed and stained with crystal violet, followed by imaging and de-staining for relative quantification.

### Statistical analysis

All values are presented as mean **±** SEM. Data were analyzed using GraphPad Prism 6 (GraphPad Software, CA, USA). Statistical significance was determined by One-way ANOVA followed by Tukey's post-test and Two-way ANOVA followed by Sidak's post-test. All the data are expressed as mean values±SEM. Where applicable, statistical significance is denoted by * for P≤0.05, ** for P≤0.01, *** for P≤0.001, and **** for P≤0.0001.

### Data and materials availability

4T1BR4 cells were obtained from Dr Normand Pouliot under the material transfer agreement. The mass spectrometry proteomics data have been deposited to the Proteome Xchange Consortium via the PRIDE partner repository with the dataset identifier PXD015141. The request for any material should be directed to Prof Kum Kum Khanna.

## Results

### Marizomib selectively inhibits proliferation of TNBC cells

Since the proteasome pathway is more prominent in TNBCs compared to other subtypes of BC and TNBCs show survival dependency on the proteasome complex 5, 10, we evaluated the anti-tumor activity of Mzb in TNBCs and other subtypes of BC. We first investigated the effect of Mzb and Btz on the CT-L, T-L, and CP-L proteasome activities in two TNBC lines, SUM159PT and MDA-MB-231. Mzb at 50 nM inhibited the CT-L, T-L, and CP-L proteasome activities by 50%, which was further reduced to 90% at 100 nM (Figure S1A). In line with a previous study 8, 100 nM Btz only inhibited the CT-L proteasome activity by 50-60% in these cells (Figure S1B). Additionally, Mzb also increased the expression of p27, a known proteasome substrate, in SUM159PT and MDA-MB-231 cells, indicating proteasome activity inhibition (Figure S1C). Thus, Mzb inhibits all three proteasome active sites in TNBC cells, exerting an overall stronger proteasome inhibitory effect in TNBCs.

**Fig 1:**
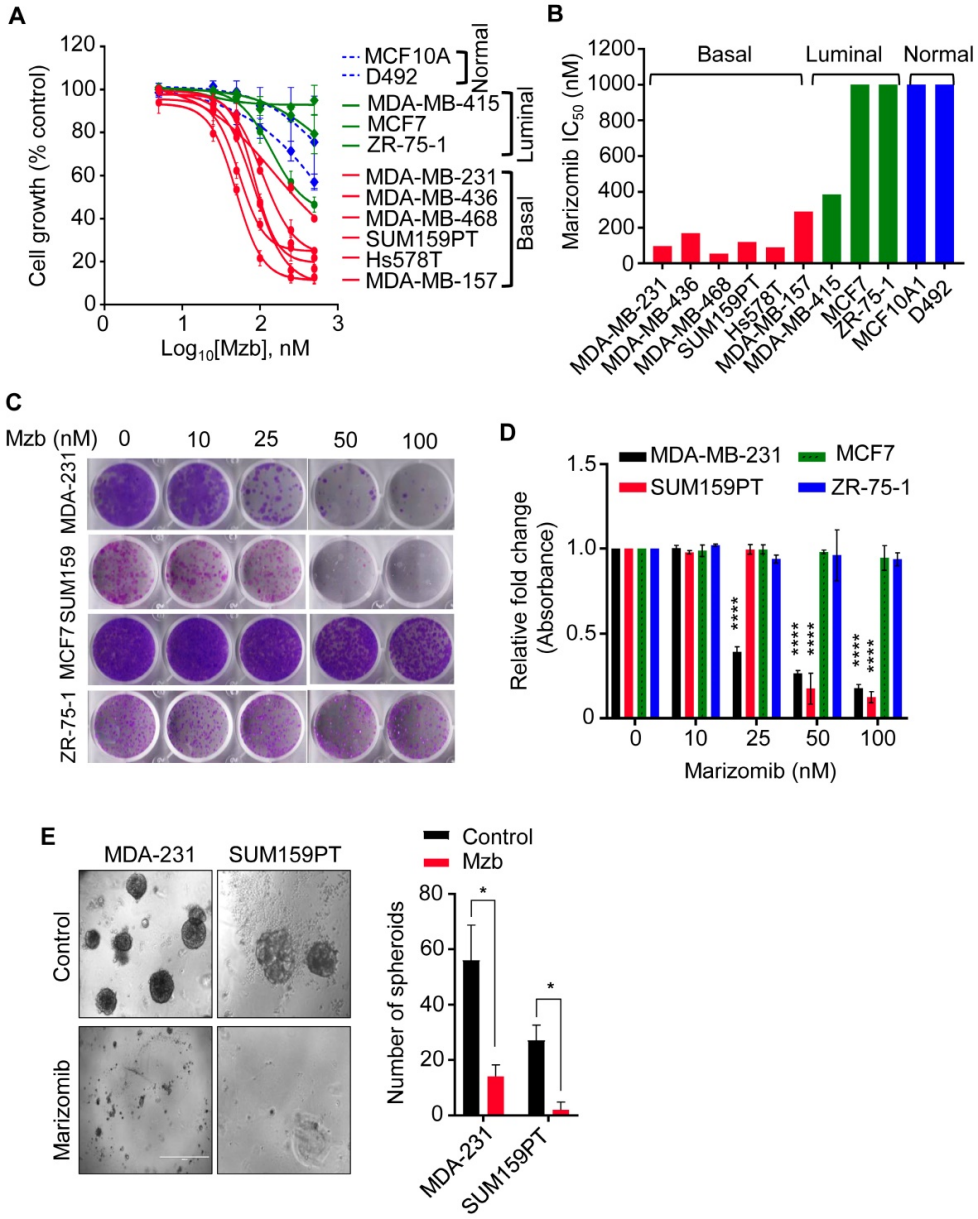
** Marizomib selectively inhibits proliferation of TNBC cells in 2D and 3D culture. (A)** A panel of TNBC (basal), luminal, and non-malignant mammary epithelial cells were treated with Mzb (0-500 nM) and cell proliferation was analyzed after 6 days using the MTS assay. The dose response curve was generated by calculating cell viability relative to the DMSO-treated control. One-way ANOVA followed by the Tukey's post-test were employed (n=3). **(B)** The IC_50_ values of marizomib in TNBC (basal), luminal, and non-malignant cells. **(C, D)** Representative images of the colony-forming capacity of TNBC lines (SUM159PT and MDA-MB-231) and luminal BC lines (MCF7 and ZR-75-1) following Mzb treatment (0-100 nM) at 14 days analyzed using crystal violet staining (C). Quantification of the colonies formed in all cell lines following Mzb treatment measured by reading crystal violet absorbance (D). One-way ANOVA followed by Tukey's post-test were employed (n=3). **(E)** Left panel, representative images of SUM159PT and MDA-MB-231 spheroids grown on Matrigel for 14 days treated with 100 nM Mzb. Right panel, quantification for a number of tumor spheroids treated with 100 nM Mzb as analyzed by counting the number of spheroids under phase-contrast microscope. The paired Student's *t* test was performed (n=3).

Mzb inhibits tumor growth in multiple cancers including myeloma, glioblastoma, leukemia, colorectal, and pancreatic cancer 17. We therefore evaluated the growth inhibitory effect of Mzb using a panel of TNBC, non-TNBC, and non-malignant mammary epithelial cell lines. Mzb selectively reduced proliferation of TNBC cells in a concentration-dependent manner (Figure 1A), without much effect on non-TNBC and non-malignant mammary epithelial cells (MCF10A and D492) (Figure 1A). The IC_50_ values for Mzb were less than 150 nM in TNBC lines and greater than 1 µM in non-TNBC lines (Figure 1B). We next analyzed the effect of Mzb on the clonogenic activity of TNBC and non-TNBC cells using a long-term colony-forming assay. Mzb significantly reduced the number of colonies for TNBC lines (SUM159PT and MDA-MB-231) but not for non-TNBC lines (MCF7 and ZR-75-1) (Figure 1C, D), suggesting that Mzb selectively inhibits TNBC cell growth.

We next examined whether Mzb can inhibit tumor growth in a 3D culture setting, which simulates the *in vivo* physiological environment of tumor cells and is used for enriching tumor-initiating cells. We used SUM159PT and MDA-MB-231 TNBC cells that form spheroids under non-adherent conditions. Mzb (100 nM) significantly reduced the size and number of MDA-MB-231 spheroids compared to untreated spheroids (Figure 1E). Interestingly, Mzb completely eliminated SUM159PT spheroid formation (Figure 1E). Thus, Mzb effectively reduces tumor cell growth in both 2D and 3D cultures.

### Marizomib induces caspase-3 dependent apoptosis in TNBC cells

Proteasome inhibitors including Btz and Mzb induce caspase-dependent apoptosis in leukemia and glioblastoma 13, 18. We therefore examined whether Mzb induces caspase-dependent apoptosis in TNBC cells. Mzb increased caspase-3 activity, as measured by the cleavage of caspase-3-specific substrate Ac-DEVD-AMC in TNBC cells but not in non-malignant breast cells (Figure S2A). Moreover, Mzb-induced increase in caspase-3 activity was rescued by co-treatment with the pan-caspase inhibitor z-VAD-FMC in SUM159PT and MDA-MB-231 (Figure S2B). Furthermore, Mzb treatment resulted in a considerable cleavage of PARP1, a classical marker of apoptosis, in TNBCs cells (SUM159PT, MDA-MB-231, and BT-549) (Figure S2C). We also analyzed the effect of Mzb treatment on the expression of various anti-apoptotic, pro-survival, and pro-apoptotic proteins. Our results showed that Mzb reduced the expression of anti-apoptotic proteins Mcl-1 and Bcl-2, and of the survival protein survivin. Notably, Mzb treatment increased the expression of pro-apoptotic protein Bim (Figure S2D). Hence, our data reveal that Mzb induces caspase-3-dependent apoptosis in TNBC cells.

### Marizomib inhibits mitochondrial respiration and OXPHOS in TNBC cells

**Fig 2:**
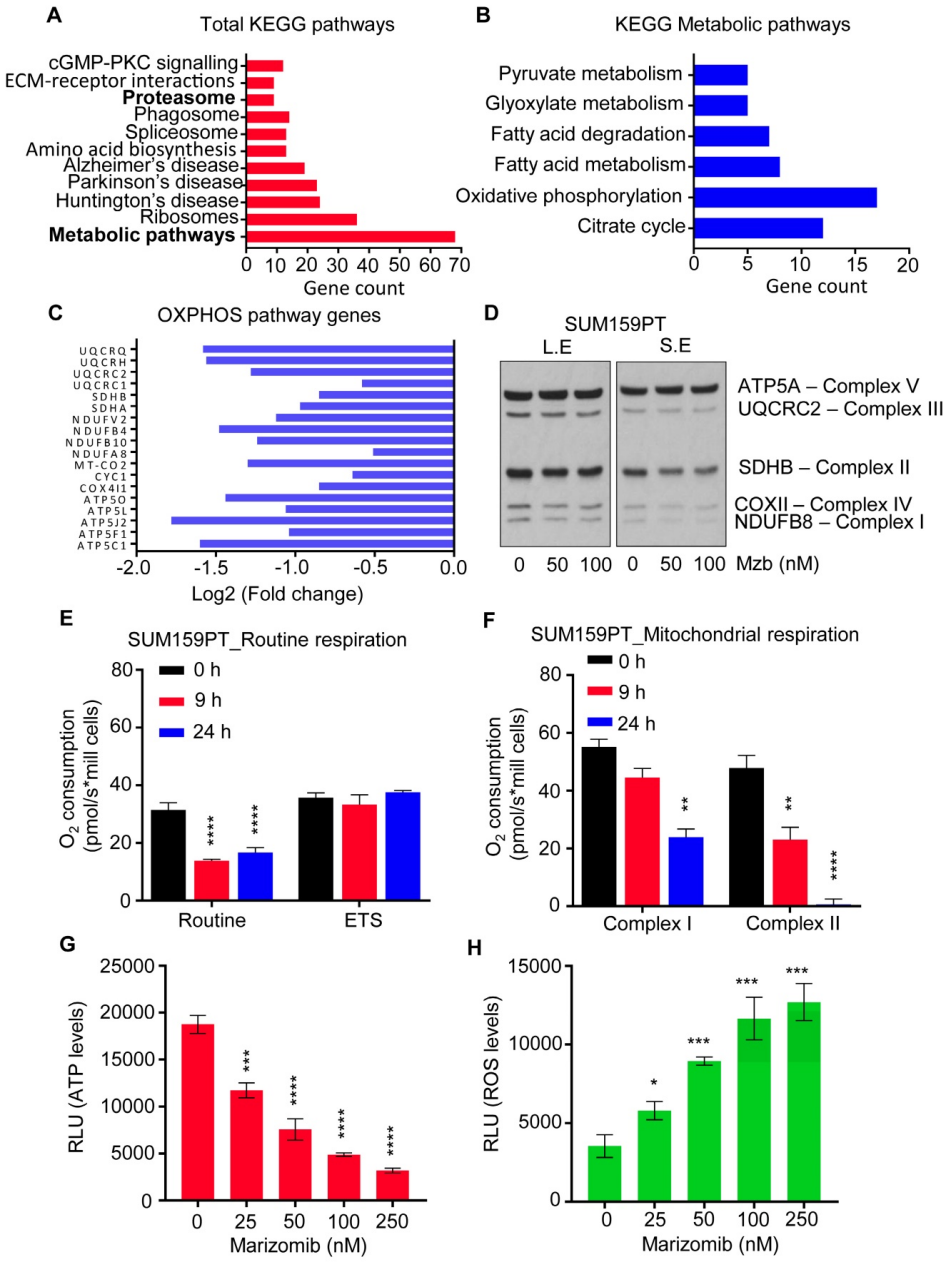
** Marizomib inhibits mitochondrial respiration and OXPHOS in TNBC cells. (A-C)** SUM159PT cells were treated with Mzb (100 nM) for 9 h and label-free global proteomic analysis was carried out. List of total KEGG pathways (A), KEGG metabolic pathways (B), and the OXPHOS pathway proteins (C) downregulated following 9 h of Mzb treatment. **(D)** SUM159PT cells were treated with Mzb (0-100 nM) for 24 h and protein levels of indicated OXPHOS pathway proteins were analyzed by western blotting. Left panel represents a long exposure (L.E) and right panel represents a short exposure (S.E). **(E, F)** SUM159PT cells were treated with Mzb (100 nM) for 9 and 24 h. (E) Oxygen consumption for ROUTINE respiration and FCCP-stimulated uncoupled respiration capacity (ETS) were evaluated on intact cells. (F) Oxygen consumption of the cells was evaluated for Complex I/CII-linked respiration. One-way ANOVA followed by Tukey's post-tests were employed (n=4 or 5). **(G, H)** SUM159PT cells were treated with Mzb (0-100 nM) for 24 h and intracellular ATP (G) and ROS (H) levels were analyzed. One-way ANOVA followed by Tukey's post-tests were employed (n=3).

Proteasome inhibitors such as Btz affect other targets in cancer cells in addition to their primary target, the proteasome complex. Hence, we investigated possible additional targets of Mzb in TNBC cells that may also contribute to the observed anti-cancer activity. To address this question, SUM159PT cells were treated with 100 nM Mzb for 0 and 9 h (before apoptosis induction) and label-free global proteomic analysis was carried out to identify the proteins or pathways altered by Mzb. In total 2547 proteins were identified out of which, 425 proteins were downregulated (log2 ≤0.6) and 293 proteins were upregulated (log2 ≥0.6). Mzb reduced the levels of 11 proteasome subunits (Figure 2A). However, we also observed upregulation of 5 proteasome subunits (PSMA5, PSMC2, PSMD2, PSMA1, PSMA7, and PSMA8) as a “bounce-back” response to cope with the loss of proteasome activity and proteotoxic stress. Interestingly, metabolism pathway components were also significantly downregulated following a 9 h Mzb treatment (Figure 2A). When we looked at individual metabolic pathways, we observed that 18 proteins of the OXPHOS pathway were downregulated (log2 ≤0.6) following Mzb treatment (Figure 2B, C). We next analyzed the protein levels of one representative component of each of the mitochondrial respiration complexes including NDUFB8 (complex I), SDHB (complex II), UQCRC2 (complex III), COX-II (complex IV), and ATP5A (complex V) by western blotting. Mzb markedly reduced SDHB and NDUFB8 protein levels (Figure 2D), suggesting that Mzb primarily affects mitochondrial complex I and II.

Although TNBCs have been shown to rely on glycolysis 19, 20, recent studies have shown that the components of the mitochondrial OXPHOS pathway are upregulated in TNBCs, and its suppression by OXPHOS inhibitors reduces TNBC tumor progression 2, 21. Hence, we further explored this novel OXPHOS inhibitory activity of Mzb in TNBCs. We evaluated routine respiration and mitochondrial respiration of SUM159PT cells following Mzb treatment using high-resolution respirometry. We found that Mzb significantly reduced routine respiration of the cells by ~50% after 9- and 24- h treatments, however the maximum respiratory capacity of the cells was unaffected (Figure 2E). In order to better understand the respiration inhibition capability of Mzb, we next examined if Mzb inhibits complex I-dependent or complex II-dependent respiration in SUM159PT cells. While 9 h Mzb treatment had no significant effect on complex I-dependent respiration, it inhibited complex II-dependent respiration by ~50% (Figure 2F). Moreover, we observed a complete inhibition of complex II-dependent respiration after 24 h Mzb treatment, but only observed a marginal inhibition of complex I-dependent respiration (Figure 2F). Furthermore, Mzb reduced ATP generation (Figure 2G) and increased reactive oxygen species (ROS) formation (Figure 2H) in SUM159PT cells in a dose-dependent manner. Similar to the effects on SUM159PT cells, Mzb reduced routine and complex II-dependent respiration (Figure S3A, B), decreased ATP generation (Figure S3C), and increased ROS levels (Figure S3D) in MDA-MB-231 cells. Hence, Mzb inhibits mitochondrial respiration in TNBC cells in addition to the proteasome complex inhibition.

Additionally, in order to compare the inhibitory effect on OXPHOS between Mzb and the FDA-approved proteasome inhibitor, bortezomib (Btz), we also tested the effect of Btz on mitochondrial respiration in SUM159PT cells. We found that, in contrast to Mzb, Btz (100 nM) has minimal inhibitory effect on routine respiration at 9 and 24 h treatment (Figure S3E). However, in contrast to Mzb, Btz treatment inhibited the maximum respiratory capability of SUM159PT cells (Figure S3E).

### Marizomib inhibits OXPHOS in a PGC-1α-dependent manner

We next delineated the mechanism by which Mzb inhibits OXPHOS in TNBC cells. Since PGC-1α, a member of PGC-1 family of transcriptional co-activators, is known to regulate OXPHOS in breast cancer, we examined the effect of Mzb on PGC-1α expression in SUM159PT cells. We found that Mzb (100 nM) markedly reduced PGC-1α protein levels within 9 h treatment (Figure S4A). The reduced levels of PGC-1α are most likely due to reduction in mRNA levels of PGC-1α after Mzb (100 nM) treatment (Figure S4B). Moreover, the mRNA levels of components of complex-I and II OXPHOS genes (SDHA, SDHB, NDUFB8, and NDUFB4) (Figure S4B), downstream targets of PGC-1α, were also reduced upon 9 h Mzb treatment. In contrast, 9 or 24 h Btz treatment (100 nM) had no effect on the PGC-1α protein levels in SUM159PT cells (Figure S4C). Btz treatment (100 nM, 24 h) only marginally reduced PGC-1α, SDHA, and NDUFB4 mRNA levels (Figure S4D), suggesting that Btz does not inhibit PGC-1α-driven OXPHOS in TNBC cells.

To further confirm the role of PGC-1α in Mzb-induced OXPHOS inhibition and cell death, we exogenously overexpressed PGC-1α in SUM159PT cells and subsequently treated them with Mzb (100 nM) for 9 h to assess OXPHOS gene expression and for 24 h to assess cell viability. Interestingly, PGC-1α overexpression partially rescued SUM159PT cells from undergoing Mzb-induced cell death (Figure S4E) and it also rescued Mzb-induced OXPHOS gene downregulation (Figure S4F). Hence, our data indicates that Mzb exerts its anti-cancer activity in-part via PGC-1α-mediated OXPHOS inhibition.

### Marizomib suppresses TNBC tumor growth *in vivo*

**Fig 3:**
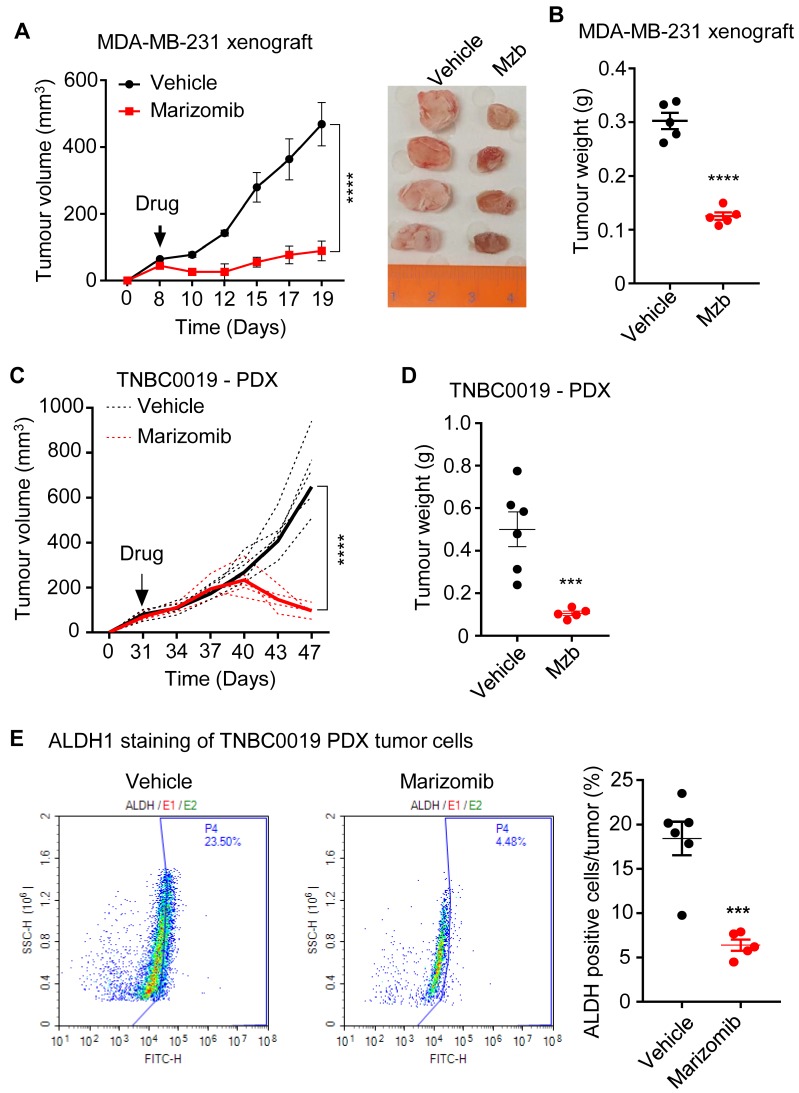
** Marizomib suppresses TNBC tumor growth *in vivo* in MDA-MB-231 xenograft and patient-derived tumor xenograft. (A, B**) MDA-MB-231 tumor growth following treatment with the vehicle or Mzb (0.15 mg/kg, twice/week, IP) for two weeks. The mean tumor size of each treatment group (A) is presented. Tumor weight (B) measured at the end of the two weeks treatment is presented (n=5 mice/group). **(C, D)** TNBC PDX tumor growth following vehicle or Mzb administration (0.15 mg/kg, twice/week, IP) 2-week treatment. The growth of individual PDX tumors (dotted line) and their mean tumor size (solid line) is presented (C). Tumor weight (D) was measured at the end of the (n=6 mice for control and n=5 mice for Mzb-treated). **(E)** PDX tumors treated with vehicle or marizomib for 2 weeks were stained with the antibody against human ALDH1, and BCSC populations were analyzed by flow cytometry (n=6 mice for control and n=5 mice for Mzb-treated). Representative images of contour plot of ALDH1^+^ (left panel). Quantification of ALDH1^+^ subpopulation of cells is presented in right panel.

Since FDA-approved peptide-based proteasome inhibitor Cfz failed to suppress tumor growth in a human breast cancer cell line MDA-MB-231-derived xenograft model 5, we evaluated the anti-cancer activity of Mzb in TNBC models *in vivo*. For this purpose, we first used MDA-MB-231 xenografts. Mzb treatment significantly reduced tumor volume (Figure 3A) and tumor weight (Figure 3B) compared to the vehicle-treated group, suggesting the superior anti-cancer activity of Mzb in TNBCs. We then analyzed the anti-tumor activity of Mzb using a TNBC patient-derived tumor xenograft (PDX). Interestingly, Mzb treatment significantly inhibited tumor growth (Figure 3C) and reduced tumor weight (Figure 3D) in our PDX model.

One of the reasons for the lack of an effective therapy for TNBC patients is the inability of the current therapies to effectively eradicate tumor-initiating cells, also known as breast cancer stem cells (BCSCs). BCSCs are inherently resistant to the standard-of-care neoadjuvant chemotherapies and radiotherapy, resulting in therapy resistance and metastasis 22-25. Since Mzb treatment significantly regressed patient-derived tumors *in vivo* (Fig 3C), we analyzed BCSC population in the tumor tissues resected at the end of a two-week treatment. The resected patient-derived control tumors (vehicle-treated) were enriched with an ALDH1^+^ population. Mzb treatment significantly reduced the percentage of ALDH1^+^ BCSCs from approximately 23% in control tumors to 4-5% (Figure 3E), suggesting that Mzb does not only impact the bulk-tumor population but also impacts BCSCs, and therefore regressed patient-derived tumors *in vivo*.

**Fig 4:**
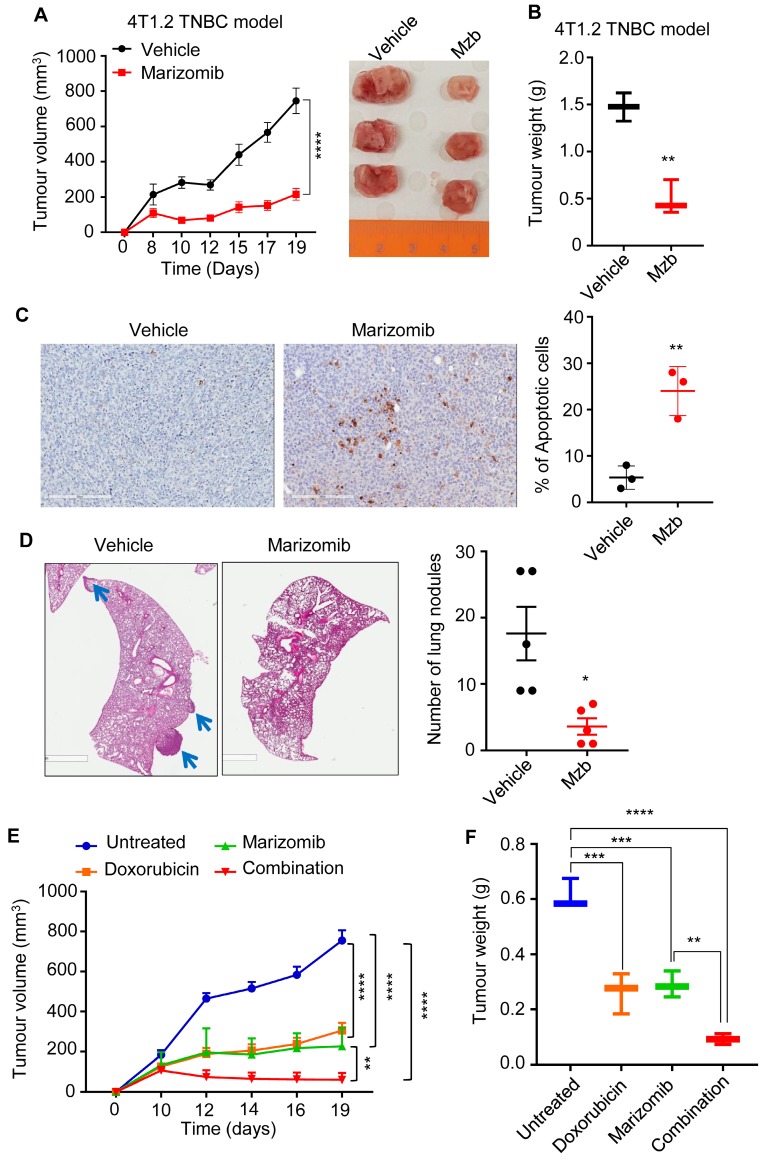
** Marizomib inhibit tumor growth in syngeneic 4T1.2 murine model of TNBC *in vivo*. (A, B)** Murine 4T1.2 syngeneic TNBC tumor growth following treatment with vehicle or Mzb (0.15 mg/kg, twice/week, IP) for two weeks is shown. Treatment was started once tumors became palpable. The mean tumor volume (A) of each treatment group and tumor weight (B) from each mouse is presented (n=6 mice/group). **(C)** Left, representative image of ApopTag staining of primary 4T1.2 tumors treated with the vehicle or Mzb for 2-weeks. Right, quantification for percentage of ApopTag-positive cells in primary 4T1.2 tumors following Mzb treatment. Unpaired “*t*” test was performed (n=3). **(D)** Left panel, representative images of lung metastasis in 4T1.2 tumor model treated with the vehicle or Mzb for two weeks. The metastatic nodules were stained with H&E. Two-way ANOVA followed by Sidak's post-test were employed for tumor growth analysis and the paired student “*t*” test was employed for tumor weight analysis and lung metastasis analysis (n=5). **(E, F)** Murine 4T1.2 syngeneic TNBC tumor growth following treatment with the vehicle, Mzb (0.075 mg/kg), doxorubicin (5 mg/kg), and combination for two weeks. The mean tumor volume (E) of each treatment group and tumor weight (F) from each mouse is presented (n=6 mice/group). Two-way ANOVA followed by Sidak's post-test for tumor growth analysis and one-way ANOVA followed by Tukey's post-tests for tumor weight analysis were performed.

We next tested the anti-cancer activity of Mzb using a fully immunocompetent murine pre-clinical syngeneic model. 4T1.2 is highly aggressive murine TNBC cell line that aptly recapitulates human TNBC tumor phenotypes after orthotopic mammary fat pad implantation in immunocompetent Balb/c mice. Our results show that Mzb significantly reduced 4T1.2 tumor growth (Figure 4A) and tumor weight (Figure 4B) compared to the vehicle-treated group. We also analyzed the percentage of apoptotic cells in vehicle- and Mzb-treated tumors by ApopTag staining and observed an increased percentage of ApopTag-positive cells in Mzb-treated 4T1.2 tumor tissues (Figure 4C), suggesting that Mzb induces apoptosis *in vivo*. Since 4T1.2 model is invasive and forms spontaneous lung metastasis, we examined whether Mzb was able to inhibit lung metastasis in the 4T1.2 model. Hematoxylin and eosin (H&E) staining of the lungs isolated from vehicle- and Mzb-treated mice after two weeks of treatment showed a significant reduction in the number of lung metastatic nodules in Mzb-treated mice compared to vehicle-treated mice (Figure 4D). We then examined the effect of Mzb on the expression of various epithelial-to-mesenchymal (EMT) markers and cell migration *in vitro*. Mzb treatment (100 nM) reduced mRNA levels of multiple EMT markers including ZEB1, Vimentin, and Slug in 4T1.2, SUM159PT, and MDA-MB-231 cells (Figure S5A-C). Mzb also reduced SUM159PT and MDA-MB-231 cell migration *in vitro* (Figure S5D). Thus, our data indicate that Mzb reduces TNBC tumor growth and inhibits metastases.

We next evaluated the anti-cancer activity of Mzb in combination with a standard-of-care chemotherapy, doxorubicin, in a syngeneic 4T1.2 model of TNBC. Mice were treated with half-maximum tolerated dose (MTD) of Mzb (0.075 mg/kg) or doxorubicin (5 mg/kg) alone or in combination. Both Mzb and doxorubicin when used as monotherapy significantly delayed tumor growth compared to untreated tumors. Notably, we observed that combination of Mzb and doxorubicin significantly inhibited 4T1.2 tumor growth (Figure 4E, F) compared to Mzb and doxorubicin monotherapy. The combination was well-tolerated as no significant weight loss was observed. Thus, our data suggests that combining Mzb with doxorubicin could serve as a better therapeutic strategy for improved clinical outcomes in TNBC patients.

### Marizomib reduces spontaneous metastasis in murine TNBC model by inhibiting EMT and circulating tumor cells

**Fig 5:**
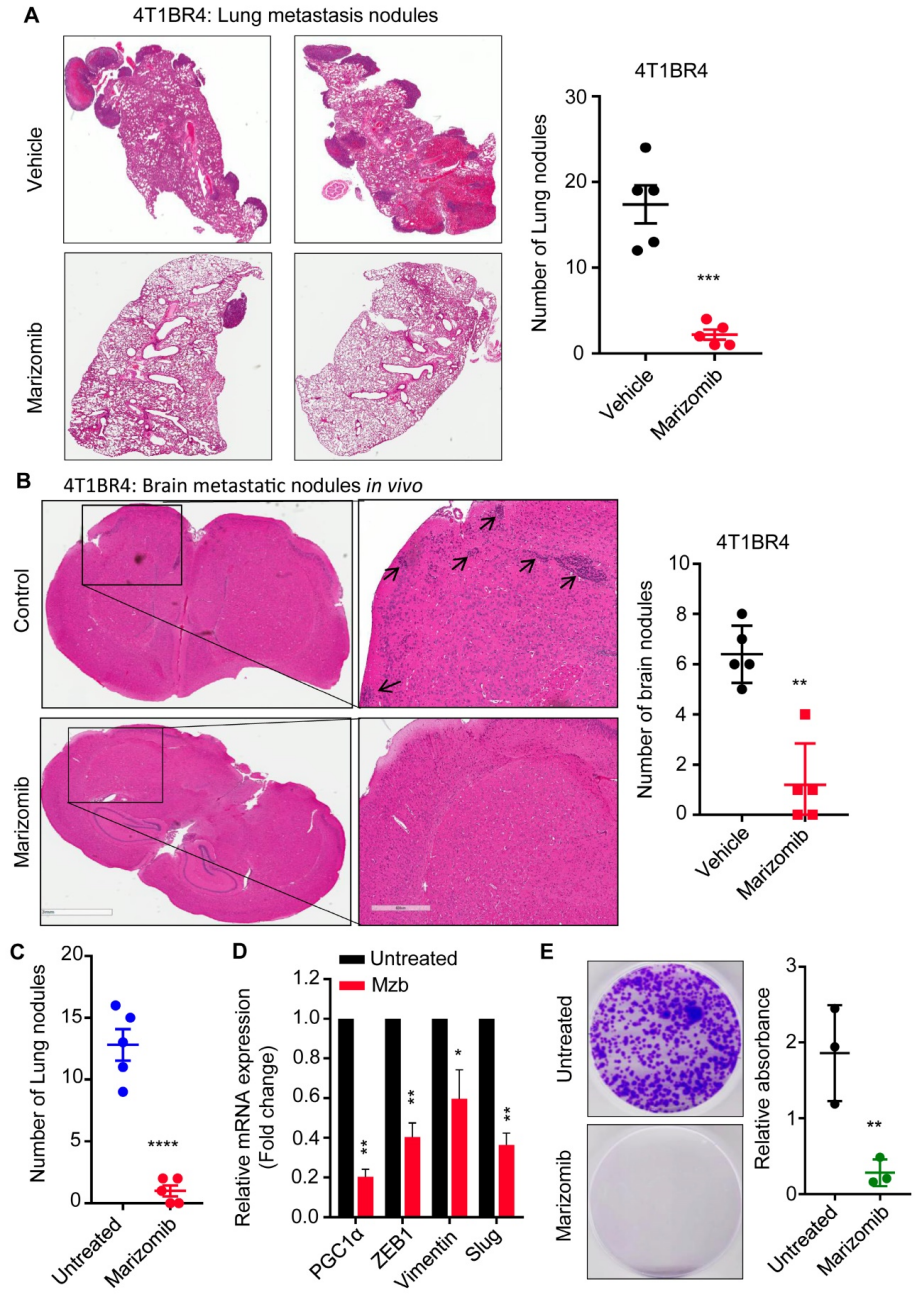
** Marizomib inhibits spontaneous lung and brain metastasis *in vivo.* (A)** Representative images of metastatic 4T1BR4 tumors in the lungs (left panel) after resecting primary tumors and following the vehicle or Mzb treatment. The metastatic nodules were stained with H&E staining (n=5 mice/group). Number of micro and macro metastatic 4T1BR4 nodules in the lungs (right panel) are shown. **(B)** Representative images of metastatic 4T1BR4 tumors in the brain (left panel) after resecting primary tumors and following vehicle or Mzb treatment. The metastatic nodules were stained with H&E staining (n=5 mice/group). Number of micro metastatic 4T1BR4 nodules in the brain (right panel) are shown (n=5 mice/group). The unpaired student “*t*” test was employed. **(C)** The number of lung metastatic nodules stained with H&E staining in primary 4T1BR4 tumors treated with the vehicle or Mzb (0.15 mg/kg) for one week. The unpaired “*t*” test was performed (n=5). **(D)** Expression levels of PGC-1α, ZEB1, Vimentin, and Slug mRNA in vehicle- and Mzb-treated primary 4T1BR4 tumors (n=3) were analyzed by RT-qPCR. **(E)** Left panel, representative images for circulating tumor cells isolated from the blood of vehicle- or Mzb-treated mice implanted with 4T1BR4 tumors. Right panel, quantification of circulating tumor cells colonies stained with crystal violet (n=3). The unpaired “*t*” test was performed.

Although Mzb inhibited lung metastasis in 4T1.2 tumor model (Fig 4D), it is possible that the effect on metastasis in this model is due to suppression of primary tumor growth (Figure 4A). To overcome the potential confounding effects on primary tumor growth, we next investigated the effect of Mzb on spontaneous or established lung and brain metastasis using a more aggressive syngeneic murine 4T1BR4 TNBC model. The 4T1BR4 cells are derived from the parental 4T1 cells with approximately 20% uptake rate in the brain and 60% uptake rate in the lungs 26. Following the engraftment of 4T1BR4 cell and once the tumor size reached ~250-300 mm^3^, we surgically resected the primary tumors. Two days after resection, mice were treated with the vehicle or Mzb (0.15 mg/kg) for two weeks. At the end of the treatment, lung and brain metastasis were analyzed. Our data revealed that Mzb dramatically reduced the number of micro and macro lung metastatic nodules in 4T1BR4 tumor model (Figure 5A). Similarly, Mzb reduced the number of micro brain metastatic nodules (Figure 5B). We were unable to assess the effect of Mzb on macro-brain metastasis as these are not evident in the 4T1BR4 model. Our data convincingly show that Mzb exerts potent cytotoxicity on metastatic TNBCs by reducing the overall lung metastasis burden and micro metastasis in the brain.

Mzb has been shown to reduce prostate tumor metastasis by suppressing the expression of EMT markers via NF-κβ inhibition 27. In our study we also found that Mzb-induced a significant downregulation of the key regulator of cell metabolism PGC-1α, which has been shown to be upregulated in metastatic breast cancer cells and to promote breast cancer metastasis by stimulating OXPHOS, increasing cell motility and invasive properties without impacting the expression of EMT-related genes 28, suggesting that Mzb catalyzed suppression of PGC-1α levels is not mutually exclusive to suppression of EMT markers. We examined the effect of Mzb on the expression of PGC-1α and EMT markers in 4T1BR4 primary tumors treated with the vehicle or Mzb for one week. This treatment significantly reduced the number of lung metastatic nodules in 4T1BR4 model *in vivo* (Figure 5C). Notably, Mzb significantly reduced the mRNA levels of PGC-1α and EMT genes including ZEB1, vimentin, and slug (Figure 5D) in primary tumors. Moreover, PGC-1α and EMT genes are known to regulate the number of CTCs, and promote breast cancer metastasis 29. Next, we examined the effect of Mzb on CTCs. We isolated CTCs from the blood of both vehicle- and Mzb-treated mice. Interestingly, Mzb dramatically reduced CTCs in the 4T1BR model *in vivo* (Figure 5E)*.* Hence, our data suggested that Mzb may inhibit TNBC lung metastasis via repressing PGC-1α and consequent OXPHOS inhibition in addition to suppressing expression of EMT markers.

Due to an established role of OXPHOS in TNBC metastasis in pre-clinical models 29, we next examined the expression of candidate OXPHOS genes in the breast cancer patient dataset GSE110590 30. We analyzed the absolute expression values of RNA derived from RNA sequence normalized counts (RSEM) from matched primary breast cancer, brain metastases and lung metastases (n=6 for lung metastases, n=5 for brain metastases). Interestingly, our results show that the expression of OXPHOS genes was significantly higher in lung and brain metastatic patients compared to their matched primary tumors (Figure S6). These data suggest that OXPHOS pathway is enriched in metastatic patients and may potentially drive metastasis in TNBC patients, and therefore Mzb could provide an effective therapy option for metastatic TNBC patients or prevent formation of metastatic tumors in patients.

### Marizomib exerts a synergistic anti-cancer activity with glycolysis inhibitor

**Fig 6:**
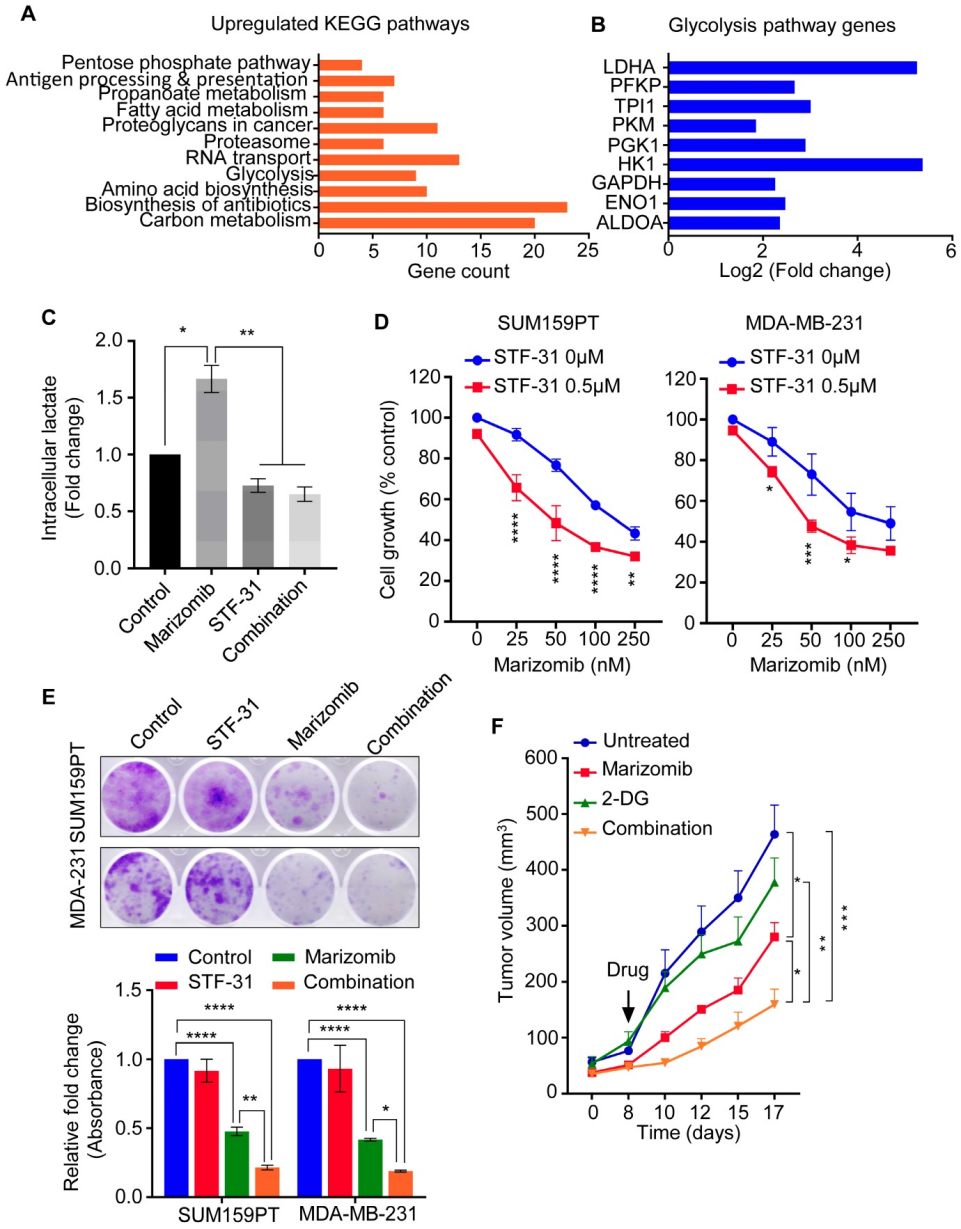
** Marizomib exerts a synergistic anti-cancer activity with glycolysis inhibitor. (A, B)** List of KEGG pathways (A) and glycolysis pathway proteins (B) upregulated in SUM159PT cells following 9 h Mzb (100 nM) treatment. **(C)** SUM159PT cells were treated with Mzb (100 nM) and STF-31 (0.5 µM), alone or in combination for 16 h. Intracellular lactate levels were analyzed by the Lactate-Glo assay kit. One-way ANOVA followed by Tukey's post-tests were employed (n=3). **(D)** SUM159PT and MDA-MB-231 cells were treated with Mzb (0-250nM) with or without STF-31 (0.5 µM) for 4 days, and cell viability was analyzed by the MTS assays. Two-way ANOVA followed by Sidak's post-test were employed (n=3). **(E)** Representative images of colony-forming capacity of SUM159PT and MDA-MB-231 cells following the treatment with Mzb (100 nM) and STF-31 (0.5 µM), both alone and in combination, at 14 days analyzed using crystal violet staining (upper panel). Quantification of the colonies formed in both cell lines measured by reading crystal violet absorbance (lower panel). One-way ANOVA followed by Tukey's post-test were employed (n=3). **(F)** Murine 4T1.2 syngeneic TNBC tumor growth following the treatment with vehicle, Mzb (0.075 mg/kg), 2-DG (400 mg/kg), and combination for two weeks. The mean tumor volume of each treatment group is presented (n=6 mice/group). Two-way ANOVA followed by Sidak's post-test were employed.

In response to OXPHOS inhibition, cancer cells switch to glycolysis to fulfill their high metabolic demand and survival 31. We therefore examined if Mzb upregulates glycolysis in TNBC cells when OXPHOS is inhibited. We analyzed our proteomic data and searched for the pathways upregulated in response to Mzb in SUM159PT cells and found a significant upregulation of two metabolic pathways, glycolysis and carbon metabolism (Figure 6A). Since glycolysis has been previously shown to compensate for the loss of the OXPHOS pathway 31, we focused on glycolysis for further analysis. We identified a significant upregulation of 9 components of the glycolysis pathway including LDHA, HK1, PGK1, and GAPDH following Mzb treatment (Figure 6B). This suggests that upregulated glycolysis may compensate for the loss of OXPHOS to fuel the metabolic demands of TNBC cells. Furthermore, Mzb treatment (100 nM) increased intracellular lactate levels in SUM159PT cells, which was reduced by STF-31 (0.5 µM), a known GLUT1 inhibitor (Figure 6C). Hence, TNBC cells upregulate glycolysis to compensate for the loss of OXPHOS following Mzb treatment.

We next examined the effect of glycolysis inhibition in combination with Mzb on TNBC cell viability. We found that Mzb exerted a synergistic growth inhibitory effect in combination with STF-31 in SUM159PT and MDA-MB-231 cells (Figure 6D). We then evaluated the effect of Mzb-STF-31 combination on the clonogenic activity of SUM159PT and MDA-MB-231 cells. Our data showed that Mzb-STF-31 combination therapy significantly reduced the number of colonies for both cell lines (Figure 6E).

We next investigated the effect of Mzb in combination with the glycolysis inhibitor *in vivo* using 4T1.2 model. For this study, we used the glycolysis inhibitor 2-deoxy D-glucose (2-DG), since STF-31 was not suitable for *in vivo* work. Our results showed that Mzb in combination with 2-DG significantly inhibited 4T1.2 tumor growth, as measured by tumor volume, compared to Mzb or 2-DG monotherapy (Figure 6F). This combination was well tolerated as indicated by the absence of weight loss (Figure S7). Notably, although Mzb monotherapy (0.075 mg/kg) significantly inhibited tumor growth, 2-DG alone had no inhibitory effect on 4T1.2 tumor growth. Collectively, our data indicate that upon OXPHOS inhibition TNBC cells switch to glycolysis, and co-inhibition of glycolysis with Mzb may provide better efficacy.

## Discussion

In this study, we show for the first time that Mzb strongly inhibits TNBC primary tumor growth and inhibits its metastasis to the lungs and micro-metastasis to the brain *in vivo.* Although TNBC cells have survival dependency on the proteasome genes and their catalytic activities 2, FDA-approved proteasome inhibitors, bortezomib and carfilzomib, showed a limited anti-cancer efficacy in the pre-clinical models of TNBC and also in clinical trials in breast cancer patients 32, 33. Initially, it was believed that poor tumor penetration was a reason for the lack of clinical activity of bortezomib in solid tumors. However, the pharmacodynamics data from two clinical trials revealed that Btz-induced proteasome inhibition is comparable in solid and blood cancer cells 34, 35. Both Btz and Cfz inhibit the CT-L activity without any significant effect on T-L and CP-L activity 12. Inhibition of CT-L by Btz or Cfz leads to a compensatory activation of T-L and CP-L, resulting in Btz/Cfz resistance in patients 10, 12. In contrast to Btz 36, 37, Mzb completely blocked CT-L, T-L, and CP-L proteasome activities in whole blood of myeloma and glioblastoma patients 12. Consistent with this, co-inhibition of multiple catalytic sites overcomes Btz resistance in the pre-clinical models of TNBCs 10. In line with these studies, we show that while Btz only inhibited the CT-L1 activity in TNBC cells, Mzb at much lower concentration inhibited all three proteasome activities and exerted a greater apoptosis in TNBC cells.

In this study, we also uncovered for the first time an additional mechanism for the anti-cancer activity of Mzb in TNBC cells. Our data demonstrates that Mzb downregulated OXPHOS proteins, inhibited complex II-dependent mitochondrial respiration, induced intracellular ROS, and inhibited mitochondrial ATP generation. Interestingly, TNBC cells have been shown to have a survival dependency on the genes of proteasome and OXPHOS pathway 2. Hence, targeting OXPHOS has emerged as a novel therapeutic strategy to combat TNBCs. In line with this notion, our data indicates that Mzb, by simultaneously inhibiting proteasome activity and OXPHOS, may provide an effective therapy option for TNBC patients.

Marizomib is currently being tested in several hematological malignancies and solid tumors both as a monotherapy, and in combination with standard-of-care chemotherapy. Mzb monotherapy reduced colorectal cancer growth *in vivo* and also sensitized the tumors to 5-fluorouracil and oxaliplatin 38. Mzb also inhibited *in vivo* tumor growth in pancreatic cancer models 39. In addition, Mzb exerted a significant anti-tumor activity in glioblastoma xenograft models, both as a single agent and in combination with HDAC inhibitors and temozolomide 13, 40. In line with these studies, our results showed that Mzb significantly reduced the tumor growth and induced apoptosis in multiple TNBC xenograft models. In contrast to Mzb, carfilzomib as a single agent showed no tumor suppressive effect in an MDA-MB-231 xenograft model 5. Mzb, when used at the half-MTD, exerted a significant anti-tumor activity in combination with doxorubicin in syngeneic murine 4T1.2 model. Thus, our data convincingly demonstrates that Mzb exerts a superior anti-cancer activity in TNBC preclinical models both as monotherapy and in combination with chemotherapy.

Cancer stem cells (CSCs) or tumor-initiating cells (TICs) play a significant role in driving tumor progression, therapy resistance, and metastasis. Usually, chemotherapies can effectively eliminate non-CSCs but lack effectiveness against CSCs, which then leads to chemoresistance and disease recurrence. It is therefore of paramount importance to eradicate stem cell populations to achieve good clinical response in patients. In breast cancer, targeting BCSCs has been shown to be an effective treatment approach as evidenced in HER2^+^ BC, where combination therapy targeting the IL6 receptor and HER2 effectively abrogated tumor growth and metastasis by eliminating BCSCs 41. In line with these observations, our study indicates that Mzb significantly targeted and reduced ALDH1^+^ BCSCs in TNBC PDX model*.* Increasing evidence suggests that mitochondrial OXPHOS is the preferred form of energy production in CSCs. Many studies have shown that CSCs, such as CD133^+^ cells of glioblastoma and pancreatic ductal adenocarcinoma, ROS^low^ quiescent leukemia stem cells, and lung and breast cancer stem cells have upregulated the OXPHOS signature and rely on the OXPHOS phenotype for their high metabolic demands 42-45. In addition, ALDH1^+^ BCSCs have been shown to have upregulated OXPHOS pathway and rely on mitochondrial respiration for survival 46. Since our data showing that Mzb inhibits OXPHOS in TNBC cells and also targets ALDH1^+^ BCSCs in PDX tumors, it can be implied that Mzb may eradicate BCSCs via OXPHOS inhibition in TNBCs. However, the detailed mechanism for the effect Mzb on mitochondrial respiration in TNBC tumor-initiating cells or ALDH1^+^ BCSCs needs further investigation.

TNBC is associated with an early risk of recurrence, high incidence of lung and brain metastasis, and overall poor survival outcomes 47, 48. The biggest clinical hurdle is how to efficiently treat metastatic TNBCs, especially brain metastasis, due to the lack of drugs capable of crossing the blood-brain barrier and exerting a potent cytotoxic activity. Mzb crosses the blood-brain barrier in glioblastoma xenograft model and exerts cytotoxic effect on tumor cells 49. Consistent with this study, our data showed that Mzb treatment significantly reduced the micro-metastatic spread of TNBCs to the brain, when tested in a murine 4T1BR4 brain-seeking syngeneic model following primary tumor resection. This is significant as current imaging modalities lack the capability of capturing micro-metastasis. Therefore, a drug that reduces even micro-metastasis formation will be important in shaping the prognosis of the patients with TNBC. These data also imply that similar to glioblastoma, Mzb can cross the blood-brain barrier, reach brain metastatic TNBC, and exert cytotoxic effect.

Our findings also show that Mzb significantly reduces lung metastasis in the aggressive and highly metastatic 4T1BR4 syngeneic TNBC model *in vivo* (Figure 5). Metastasis is thought to be driven by invading cancer cells or CTCs. CTCs rely on OXPHOS for their energy supply during their transit to the distant organ; such OXPHOS is stimulated by increased PGC-1α expression 29. PGC-1α downregulation reduced lung metastasis by reducing the expression of various OXPHOS genes without any significant effect on EMT genes 29, suggesting that in breast cancer settings PGC-1α may drive metastasis independent of EMT. Molecular analysis of primary 4T1BR4 tumors revealed that Mzb reduced PGC-1α mRNA levels and also significantly reduced CTCs *in vivo* in 4T1BR4 model. These data may imply that Mzb inhibits PGC-1α-stimulated OXPHOS in TNBC cells resulting in reduction in invading cancer cells or CTCs and reduced metastasis. However, further studies are required to specifically measure mitochondrial respiration and OXPHOS pathway components in CTCs isolated from Mzb-treated mice. Since we barely detected any CTCs in Mzb-treated mice, we were unable to measure mitochondrial respiration in our experiment. Moreover, our data show that Mzb also reduced mRNA levels of various EMT markers including ZEB1, Vimentin, and Slug, suggesting that Mzb may inhibit TNBC lung metastasis via multiple mechanisms.

In conclusion, our pre-clinical data generated in this study using a range of *in vitro* and *in vivo* models provide a strong rationale to translate Mzb into a potential clinical trial in combination with standard-of-care chemotherapy such as doxorubicin, and also with glycolysis inhibitors for TNBC patients. Notably, our data also show that Mzb has potential to reduce lung and brain metastatic burden in TNBC patients and hence can be translated into a clinical trial for TNBC patients in metastatic settings.

## Supplementary Material

Supplementary materials and methods, figures, and tables.

## Abbreviations

BCSCs: breast cancer stem cells
Btz: bortezomib
Cfz: carfilzomib
CP-L: caspase-like
CTCs: circulating tumor cells
CT-L: chymotrypsin-like
EMT: epithelial-to-mesenchymal transition
Ixz: ixazomib
Mzb: marizomib
OXPHOS: oxidative phosphorylation
ROS: reactive oxygen species
T-L: trypsin-like
TNBC: triple-negative breast cancer
