# Supplementary material for: Marizomib suppresses triple-negative breast cancer via proteasome and oxidative phosphorylation inhibition
Source: Theranostics. 2020 Apr 6;10(12):5259–75. doi: 10.7150/thno.42705 (PMC7196287; doi:10.7150/thno.42705)
Supplement: Supplementary file 1 — Supplementary materials and methods, figures, and tables. [file thnov10p5259s1.pdf]

Supplementary Files:

Materials & Methods:

Proteasome activity assays:

The CT-L, T-L, and CP-L proteasome activities were measured using the Proteasome-Glo™ Assays Kit (Promega) as per the manufacturer's guidelines.

Cell viability assays:

The breast cancer cells and non-malignant mammary epithelial cells were treated with Mzb for 6 days in 48-wells plate, and cell viability was analyzed by the CellTiter 96® AQueous One Solution Cell Proliferation Assay (MTS) (Promega) as per the manufacturer's guidelines.

Colony formation assays:

The cells were treated with or without Mzb for 24 h and the effect of Mzb on a long-term colony formation capacity was analyzed as described previously (1).

3D spheroids assays:

The 3D tumor spheroid assays were performed using well established techniques as described previously (2). At the end of the treatment, images of tumor spheroids were taken by EVOS® Digital Inverted Microscope (AMG, WA, USA).

Caspase-3 activity assays:

Caspase-3 activity within the treated and untreated breast cancer cells and non-malignant mammary epithelial cells was determined as described previously following the cleavage of Ac-DEVD-AMC (Enzo Life Sciences, NY, USA), a caspase-3 substrate (3).

Immunoblotting:

Immunoblotting was performed as described previously (4), and target proteins were probed with antibodies listed in Table S1. The Super Signal chemiluminescent ECL-plus (Amersham) was used for detecting target proteins.

ATP, ROS, and intracellular lactate measurement:

For ATP levels, cells were treated with Mzb for 24 h in 96-wells plate, and intracellular ATP was determined using the CellTiter-Glo® Luminescent assay (Promega). For ROS levels, cells were treated with Mzb for 24 h in 96-wells plate, and intracellular ROS levels were analyzed by the ROS-Glo™ H_2O_2 assay kit (Promega). For assessing the intracellular lactate, cells were treated with Mzb for 16 h in 96-wells plate and intracellular lactate levels were analyzed by the Lactate-Glo™ assay (Promega).

Reverse transcriptase–quantitative PCR:

Gene expression analysis using reverse-transcriptase quantitative PCR (RT-qPCR) was performed using the LightCycler 480 (Roche, Basel, Switzerland) as described previously (5). The list of primers used is listed in Table S2.

Plasmid DNA transfection:

SUM159PT cells were transfected with either pcDNA4 empty vector or pcDNA4-Myc-PGC-1α plasmid using Lipofectamine 3000 (Promega) as per the manufacturer's guidelines. The pcDNA4-myc-PGC-1α was a gift from Toren Finkel (Addgene plasmid # 10974; http://n2t.net/addgene:10974; RRID: Addgene_10974) (6).

***In vitro* migration assays:**

The migration assays were performed using Corning® Transwell Inserts. Cells were treated with or without 100 nM Mzb for 24 h and washed. 1 X 10^5 cells were re-suspended in 0.1% FBS containing DMEM media and were seeded in the upper compartment of the inserts. In the bottom compartment, 10% FBS containing DMEM media was placed as a chemo-attractant. Cells were incubated for 24 h and the migrated cells were fixed with 0.05% crystal violet for 30 minutes and imaged.

Immunohistochemistry:

Immunohistochemical analysis was performed using primary 4T1.2 tumors as described previously (5). ApopTag staining was performed using the ApopTag peroxidase *in situ* apoptosis detection kit (S7100; Millipore-Sigma, Billerica, MA, USA).

Proteomic analysis:

All chemicals and solvents are of LCMS grade and were purchased from Merck, USA, unless stated otherwise. All plastic consumables were purchased from Eppendorf, Germany.

1. *In-solution protein digestion*

Total protein was extracted using 2%SDS protein lysis buffer. Sample protein concentrations were determined using the Pierce BCA protein assay kit (Thermo Fischer, USA), following the kit instructions. 30 µg of each protein sample (n=12) was subjected to reduction, alkylation and trypsin-protein precipitation, prior to in-solution digestion. Briefly, the native disulfide bonds in the protein samples were sequentially reduced and alkylated with 10 mM tris (2-carboxyethyl)phosphine (60°C,30 min), and 40 mM 2-chloroacetamide (dark, RT, 30 min) respectively. Following this, 10 volumes of chilled 100% methanol was added to the sample to facilitate protein co-precipitation along with trypsin (1:100 enzyme:protein; sequencing grade porcine, Promega, USA). After incubating at -20°C for 24 h, samples were centrifuged at 16,000 g for 15 min at 4 °C to collect the protein pellet. The pellet was washed multiple times with chilled methanol to remove residual detergent from the lysis buffer. Trypsin (1:100, enzyme:

protein) is added to the protein pellet and then the tube was incubated at 37°C for 18 h. After acidification to a final concentration of 1% v/v formic acid (FA); samples were desalted on the Strata X-33 µm reverse phase solid phase extraction resin (Phenomenex, USA), dried down and resuspended in 12 µl of 0.1 % v/v triflouoroacetic acid (TFA). Prior to LC-MS/MS, the digested peptide concentration was determined using the Pierce µBCA protein assay kit (Thermo Fischer, USA), following the kit instructions.

2. Tandem liquid chromatography - mass spectrometry (LC-MS/MS) and database search.

One µg digested peptides were analyzed using a Thermo Scientific VelosPro Orbitrap mass spectrometer (Thermo Fisher, USA) coupled to a Shimadzu Prominence Nano HPLC (Shimadzu, Kyoto, Japan). 5 µl of injected samples were separated on a ProteCol C18 analytical column [(150 mm x 150 µm, 3 µm; connected to a ProteCol guard column, 10 mm x 300 µm, 3 µm), Trajan Bioscience, Australia] over a gradient of 120 min at a flow rate of 1 µl / min. The peptides were eluted using Buffer A (0.1% v/v FA in water) and Buffer B (80% v/v acetonitrile in 0.1% v/v FA), over the specified gradient for 120 min (5.5% B to 40% B at 100 min). The column was maintained at 45 °C. Data acquisition on the mass spectrometer was done using the Data Dependent Top 15 method. The MS spectra were acquired in the mass range = m/z 380 — 1700 (Orbitrap resolution = 60000). Fragmentation for the MS/MS spectra were acquired using collision induced dissociation (CID) in the ion trap mode (dynamic exclusion was set at 90.00 sec).

The extracted raw data was searched for protein IDs against the reviewed human proteome database (49,070 Swissprot entries; database accessed on 01/07/2018) using MaxQuant[1] software, v. 1.5.8.3. MaxQuant parameters were set as follows: digestion = trypsin, with 2 missed cleavages; fixed modification was set to carbamidomethyl; variable modifications = none; LFQ= enabled with minimum ratio count set to 2; match between runs = true; unique and razor peptides were used for protein identification, with minimum unique peptide = 1. The generated protein list was manually filtered to remove contaminants and reverse identified protein IDs; a cut-off score of 2 for minimum peptide counts per protein was applied. Summed intensity-based normalization was carried out. A fold change (FC) cut-off of 1.5 was used to identify differentially regulated proteins (Overexpressed = $\log_2 FC \geq 0.6$; Downregulated = $\log_2 FC \leq 0.6$). Functional annotation and pathway enrichment analysis of differentially regulated proteins was performed using default parameters of DAVID.

Supplementary Figures:

Figure S1:

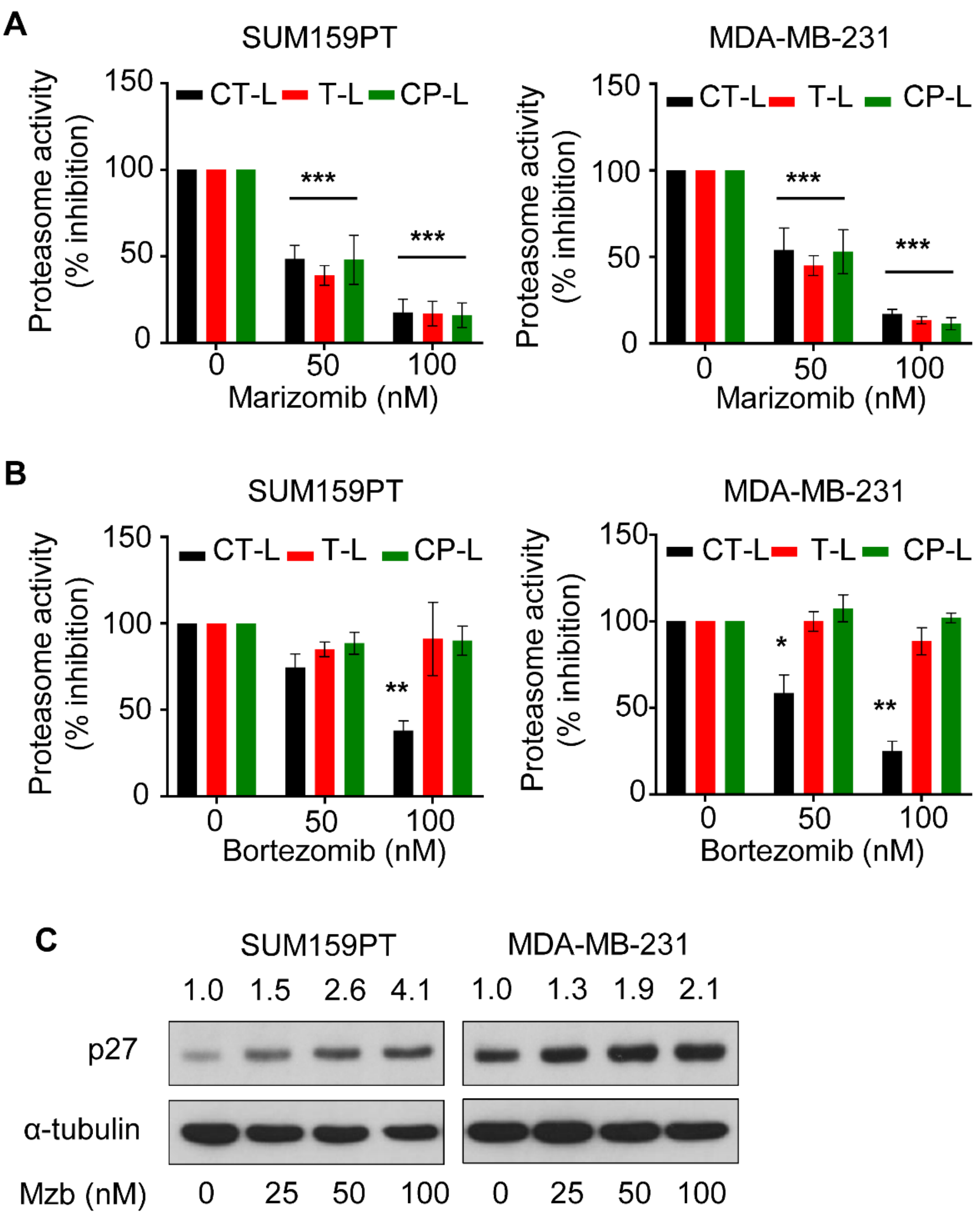

Fig S1: Marizomib inhibits CT-L, T-L, and CP-L proteasome activity in TNBC cells.

(A, B) SUM159PT and MDA-MB-231 were treated with indicated concentrations of marizomib (0-100 nM) (A) and bortezomib (0-100 nM) (B) for 24 h. The chemotrypsin-like (CT-L), trypsin-

like (T-L), and caspase-like (CP-L) proteasome activities were analyzed using Proteasome-Glo® Assays. The activity was calculated relative to untreated control (n=3).

(C) Protein levels of proteasome substrate p27 in SUM159PT and MDA-MB-231 cells following Mzb treatment (0-100 nM, 24 h).

Figure S2:

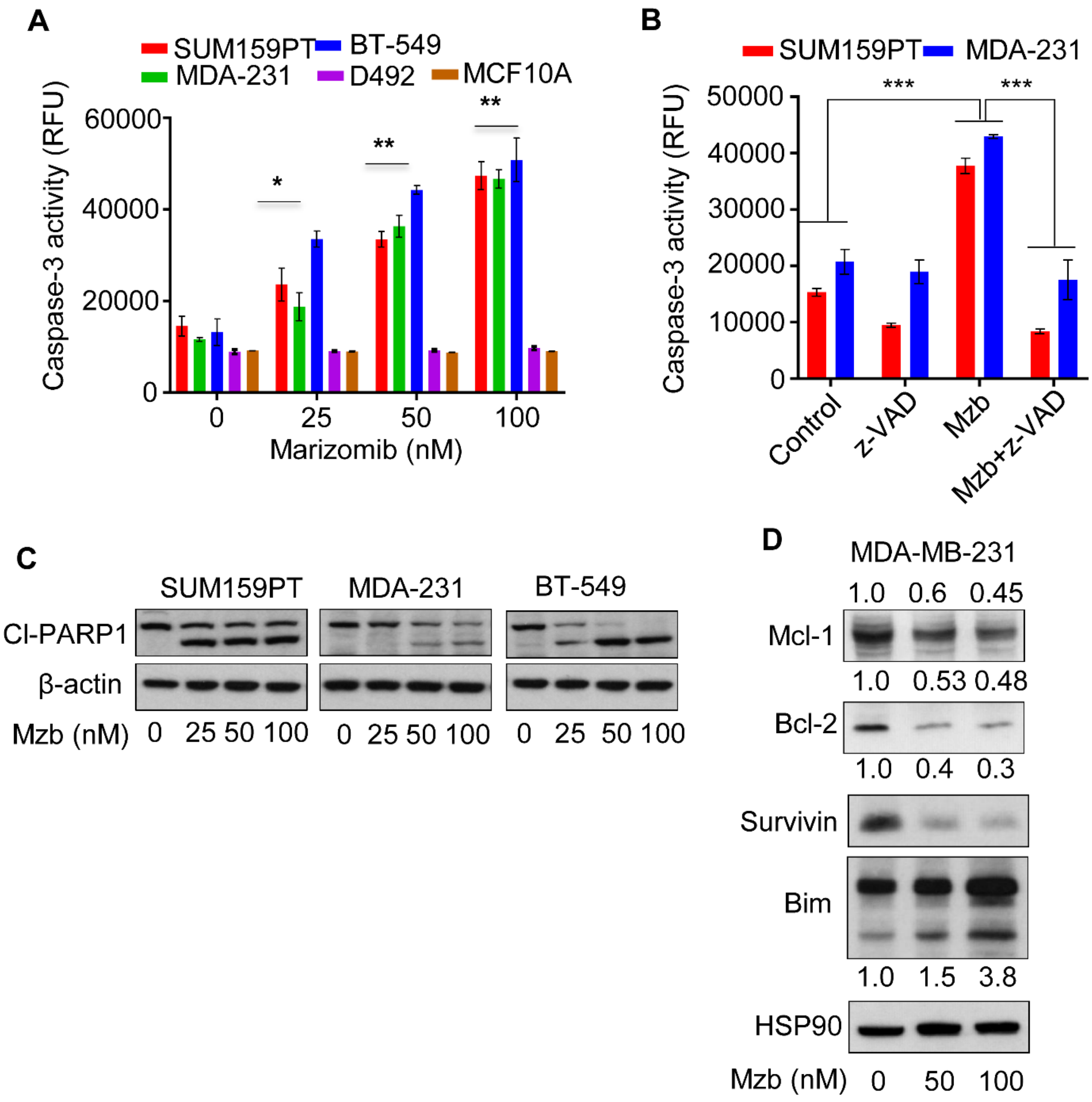

Fig S2: Mzb induces caspase-3-dependent apoptosis in TNBC cells.

(A) Indicated TNBC, non-TNBCs, and non-malignant cell lines were treated with Mzb (0-100 nM) for 24 h. Caspase-3 activity was analyzed by measuring the cleavage of Caspase-3-specific substrate Ac-DEVD-AMC (n=3).

(B) SUM159PT and MDA-MB-231 cells were pre-treated with a pan-Caspase inhibitor z-VAD-FMC (50 μM) for 2 h, and subsequently treated with or without Mzb (100 nM) for additional 24 h. Caspase-3 activity was determined by measuring Ac-DEVD-AMC cleavage. One-way ANOVA followed by Tukey's post-tests were employed (n=3).

(C) TNBC lines (SUM159PT, MDA-MB-231, and BT-549) and near-normal mammary epithelial lines (MCF10A and D492) were treated with Mzb (0-100 nM) for 24 h. The protein levels of cleaved PARP1 were analyzed by western blotting. β-actin was used as a loading control.

(D) MDA-MB-231 cells were treated with Mzb (0-100 nM) for 24 h and protein levels of indicated anti- and pro-apoptotic proteins were analyzed by western blotting. HSP90 was used as a loading control.

Figure S3:

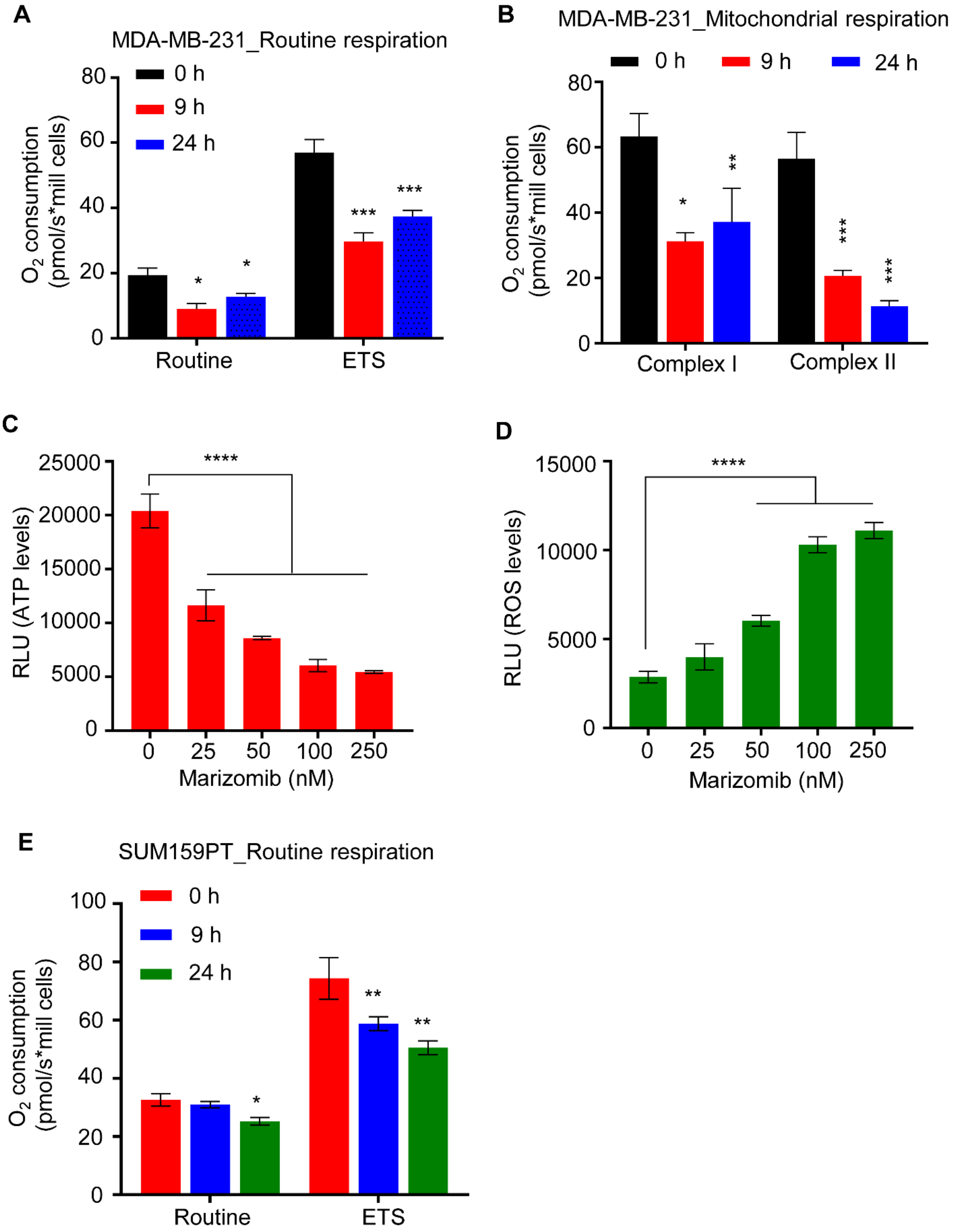

A
MDA-MB-231_Routine respiration
0 h
9 h
24 h
O2 consumption (pmol/s*mill cells)
80
60
40
20
0
*
*
***
***
Routine
ETS
B
MDA-MB-231_Mitochondrial respiration
0 h
9 h
24 h
O2 consumption (pmol/s*mill cells)
80
60
40
20
0
*
**
***
***
Complex I
Complex II
C
RLU (ATP levels)
25000
20000
15000
10000
5000
0
****
0
25
50
100
250
Marizomib (nM)
D
RLU (ROS levels)
15000
10000
5000
0
****
0
25
50
100
250
Marizomib (nM)
E
SUM159PT_Routine respiration
0 h
9 h
24 h
O2 consumption (pmol/s*mill cells)
100
80
60
40
20
0
*
**
**
Routine
ETS

Fig S3: Marizomib inhibits OXPHOS in TNBC cells.

(A) MDA-MB-231 cells were treated with Mzb (100 nM) for 9 h and 24 h. Oxygen consumption for ROUTINE respiration and FCCP-stimulated uncoupled respiration capacity (ETS) were evaluated on intact cells (n=4 or 5).

(B) MDA-MB-231 cells were treated with Mzb (100 nM) for 9 h and 24 h. Oxygen consumption of the cells was evaluated for Complex I/CII-linked respiration (n=4 or 5).

(C, D) MDA-MB-231 cells were treated with Mzb (0-100 nM) for 24 h and intracellular ATP (C) and ROS levels (D) were analyzed. One-way ANOVA followed by Tukey's post-tests were employed (n=3).

(E) SUM159PT cells were treated with Btz (100 nM) for 9 h and 24 h. Oxygen consumption for ROUTINE respiration and FCCP-stimulated uncoupled respiration capacity (ETS) were evaluated on intact cells (n=4).

Figure S4:

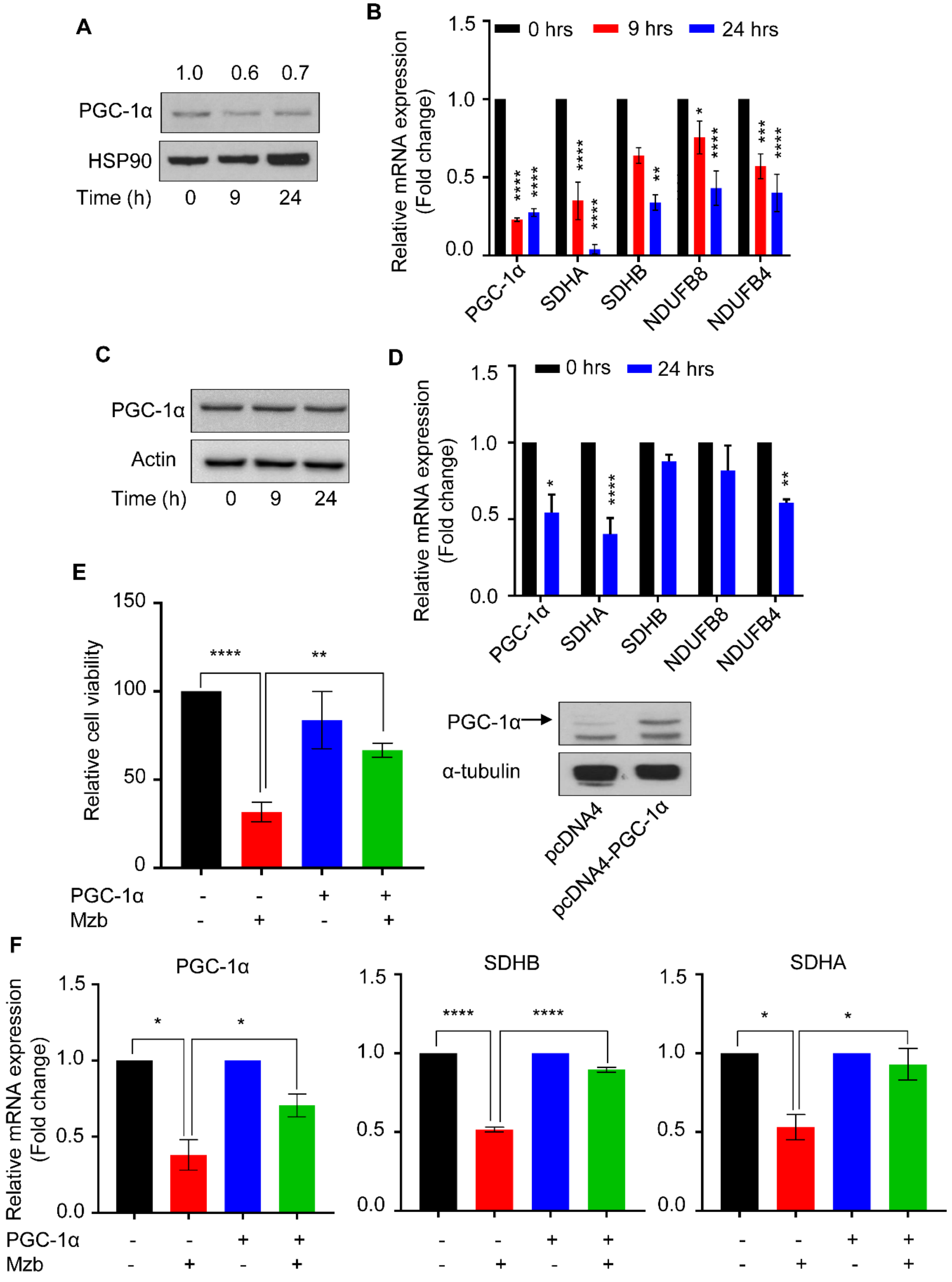
A
1.0
0.6
0.7
PGC-1α
HSP90
Time (h)
0
9
24
B
0 hrs
9 hrs
24 hrs
Relative mRNA expression (Fold change)
1.5
1.0
0.5
0.0
PGC-1α
SDHA
SDHB
NDUFB8
NDUFB4
C
PGC-1α
Actin
Time (h)
0
9
24
D
0 hrs
24 hrs
Relative mRNA expression (Fold change)
PGC-1α
SDHA
SDHB
NDUFB8
NDUFB4
E
Relative cell viability
150
100
50
0
PGC-1α
Mzb
PGC-1α
α-tubulin
pcDNA4
pcDNA4-PGC-1α
F
PGC-1α
SDHB
SDHA
Relative mRNA expression (Fold change)
PGC-1α
Mzb

Fig S4: Marizomib inhibits OXPHOS in a PGC-1α-dependent manner.

(A) SUM159PT cells were treated with 100 nM Mzb for 0, 9, and 24 h, and PGC-1α protein levels were analyzed. HSP90 was used as a loading control.

(B) SUM159PT cells were treated with 100 nM Mzb for indicated time-points, and mRNA levels of the indicated genes were analyzed by RT-qPCR. One-way ANOVA and Tukey's post were used (n=3).

(C) SUM159PT cells were treated with 100 nM Btz for 0, 9, and 24 h, and PGC-1α protein levels were analyzed. HSP90 was used as a loading control.

(D) SUM159PT cells were treated with 100 nM Btz for 24 h, and mRNA levels of the indicated genes were analyzed by RT-qPCR. One-way ANOVA and Tukey's post were used (n=3).

(E, F) SUM159PT cells were transfected with either pcDNA4 vector or pcDNA4-Myc-PGC-1α overexpression plasmid for 24 hours, and subsequently treated with 100 nM Mzb for 9 (for OXPHOS gene expression) and 24 h (for cell viability). (C) Left panel, cell viability was analyzed by Trypan blue exclusion assays. (D) OXPHOS gene expression levels were analysed by RT-qPCR. One-way ANOVA followed by Tukey's post-test were performed (n=2). Values represent mean±SD. Right panel, representative western blot images showing PGC-1α overexpression.

Figure S5:

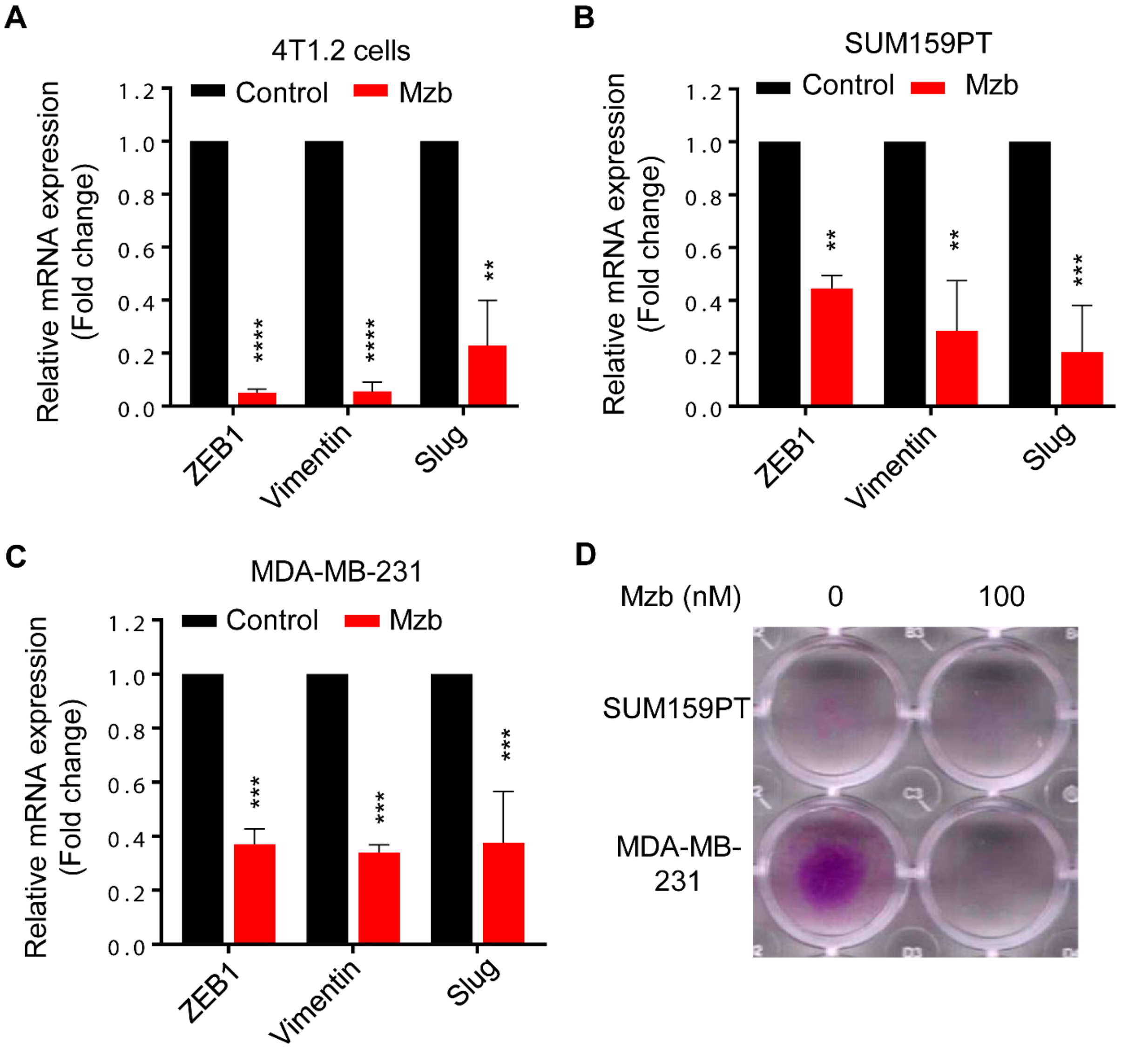

Fig S5: Marizomib reduces the expression of EMT markers and TNBC cell migration:

(A-C) 4T1.2 (A), SUM159PT (B), and MDA-MB-231 (C) cells were treated with Mzb (100 nM) for 8 h, and mRNA levels of ZEB1, Vimentin, and Slug were analyzed by RT-qPCR. The unpaired "*t*" test was performed.

(D) SUM159PT and MDA-MB-231 cells were treated with Mzb (100 nM) for 4 h, and cell migration was analyzed by the Transwell migration assay. Representative images of two independent experiments are shown.

Figure S6:

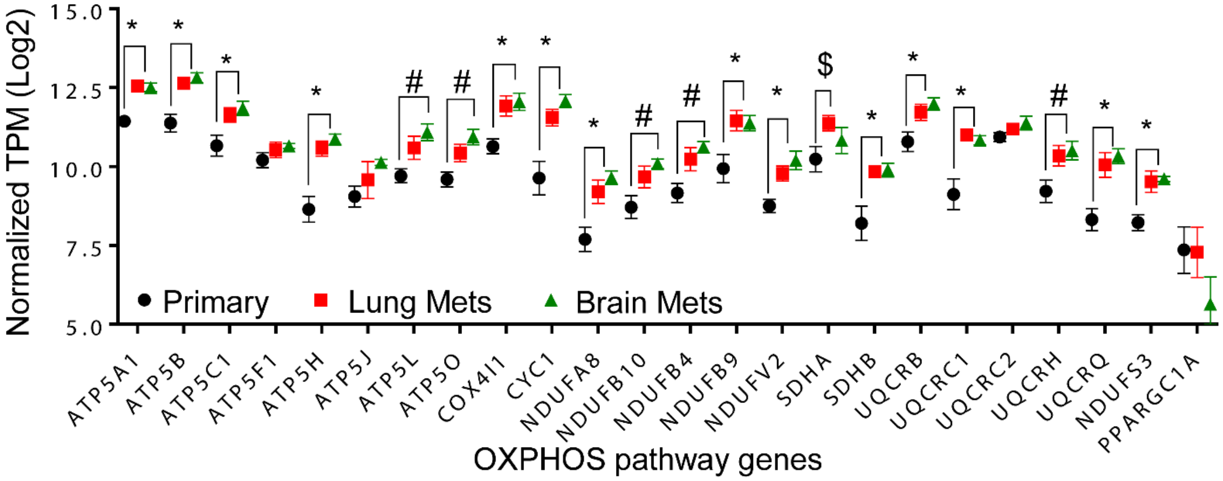

Fig S6: Expression of OXPHOS genes in breast cancer patients.

Expression of an indicated OXPHOS genes in matched primary, lung and brain metastatic breast cancer patients (n=12). The unpaired "*t*" test was performed. * (primary Vs lung and brain metastasis), (primary Vs lung metastasis), and $ (primary Vs brain metastasis).

Figure S7:

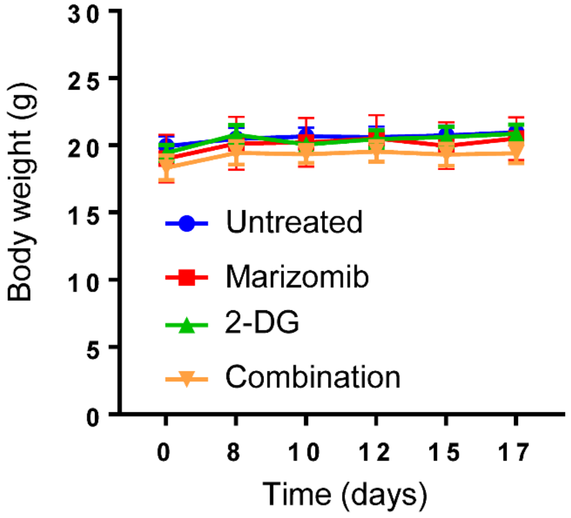

Fig S7: Effect of marizomib and 2-DG combination therapy on mouse body weight. Murine 4T1.2 syngeneic TNBC tumor growth following the treatment with vehicle, Mzb (0.075 mg/kg), 2-DG (400 mg/kg), and combination for two weeks. The mean tumor weight of each treatment group is presented (n=6 mice/group).

Supplementary Table S1:

**Antibody**	**Supplier**	**Catalogue number**
Anti-Mcl-1	Abcam	ab32087
Anti-Bcl-2	Cell signaling technology	3498T
Anti-Bim	Cell signaling technology	2933T
Survivin	Cell signaling technology	2808T
Anti-β-actin	BD Transduction Laboratories	612656
Anti-PAPR1	Cell signaling technology	9542S
Anti-OXPHOS cocktail	Abcam	ab110411
Anti-PGC-1α	Cell signaling technology	2178S
Anti-HSP90	Santa Cruz	sc-69703

Supplementary Table S2:

Gene name	Forward primer	Reverse primer
Ms PGC-1α	ACGTCCCTGCTCAGAGCTT	CCTTGGGGTCATTTGGTGAC
Ms ZEB1	CAACAAGACACCGCCGTCAT	GAGCAGCTGAAGTTGTCCTC
Ms Vimentin	GGATCAGCTCACCAACGACA	CTGCAGCTCCTGGATCTCTT
Ms Slug	TGTCTGCAAGATCTGTGGCAA	GAAGCGACATTCTGGAGAAGG
Hu PGC-1α	CCTGCATGAGTGTGTGCTCT	TGGGGTCATTTGGTGACTCTG
Hu SDHA	GGATGTCGTGGAGAGGGAGGCATT	GGTGCAGCTGCAGGTAGACGTGAT
Hu SDHB	TAGCACCAGCTGCCCCAGCTACT	CCCTGGATTCAGACCCTTAGGACA
Hu NDUFB8	CCCCACACCTGTTTCTTGGCATGT	CCACGAAGCCTCCTCAGATCTCAT
Hu NDUFB4	TCGACCCAGCCGAATACAAC	GCAGGATTTTCGATGAGCCC
Hu RPL32	CAGGGTTCGTAGAAGATTCAAGGG	CTTGGAGGAAACATTGTGAGCGATC
Hu ZEB1	GCAGCTGACTGTGAAGGTGT	CTGTACATCCTGCTTCATCTG
Hu Vimentin	CGTGTATGCCACGCGCTCCT	TCGAGCTCGGCCAGCAGGAT
Hu Slug	GCACATCCGAAGCCACAC	GGAGAAGGTCCGAGCACAC

References:

1. Kalimutho M, Sinha D, Jeffery J, Nones K, Srihari S, Fernando WC*, et al.* CEP55 is a determinant of cell fate during perturbed mitosis in breast cancer. EMBO molecular medicine **2018**;10(9) doi 10.15252/emmm.201708566.
2. Debnath J, Muthuswamy SK, Brugge JS. Morphogenesis and oncogenesis of MCF-10A mammary epithelial acini grown in three-dimensional basement membrane cultures. Methods **2003**;30(3):256-68.
3. Raninga PV, Di Trapani G, Vuckovic S, Bhatia M, Tonissen KF. Inhibition of thioredoxin 1 leads to apoptosis in drug-resistant multiple myeloma. Oncotarget **2015**;6(17):15410-24 doi 10.18632/oncotarget.3795.
4. Karlenius TC, Shah F, Di Trapani G, Clarke FM, Tonissen KF. Cycling hypoxia up-regulates thioredoxin levels in human MDA-MB-231 breast cancer cells. Biochemical and biophysical research communications **2012**;419(2):350-5 doi 10.1016/j.bbrc.2012.02.027.
5. Sinha D, Kalimutho M, Bowles J, Chan AL, Merriner DJ, Bain AL*, et al.* Cep55 overexpression causes male-specific sterility in mice by suppressing Foxo1 nuclear retention through sustained activation of PI3K/Akt signaling. FASEB journal : official publication of the Federation of American Societies for Experimental Biology **2018**;32(9):4984-99 doi 10.1096/fj.201701096RR.
6. Ichida M, Nemoto S, Finkel T. Identification of a specific molecular repressor of the peroxisome proliferator-activated receptor gamma Coactivator-1 alpha (PGC-1alpha). J Biol Chem **2002**;277(52):50991-5 doi 10.1074/jbc.M210262200.
